# Ecological genomics of local adaptation in *Cornus florida* L. by genotyping by sequencing

**DOI:** 10.1002/ece3.2623

**Published:** 2016-12-20

**Authors:** Andrew L. Pais, Ross W. Whetten, Qiu‐Yun (Jenny) Xiang

**Affiliations:** ^1^ Department of Plant and Microbial BiologyNorth Carolina State UniversityRaleighNCUSA; ^2^ Department of ForestryNorth Carolina State UniversityRaleighNCUSA

**Keywords:** *Cornus florida*, genotyping by sequencing, local adaptation, single nucleotide polymorphisms

## Abstract

Discovering local adaptation, its genetic underpinnings, and environmental drivers is important for conserving forest species. Ecological genomic approaches coupled with next‐generation sequencing are useful means to detect local adaptation and uncover its underlying genetic basis in nonmodel species. We report results from a study on flowering dogwood trees (*Cornus florida L*.) using genotyping by sequencing (GBS). This species is ecologically important to eastern US forests but is severely threatened by fungal diseases. We analyzed subpopulations in divergent ecological habitats within North Carolina to uncover loci under local selection and associated with environmental–functional traits or disease infection. At this scale, we tested the effect of incorporating additional sequencing before scaling for a broader examination of the entire range. To test for biases of GBS, we sequenced two similarly sampled libraries independently from six populations of three ecological habitats. We obtained environmental–functional traits for each subpopulation to identify associations with genotypes via latent factor mixed modeling (LFMM) and gradient forests analysis. To test whether heterogeneity of abiotic pressures resulted in genetic differentiation indicative of local adaptation, we evaluated *F*_st_ per locus while accounting for genetic differentiation between coastal subpopulations and Piedmont‐Mountain subpopulations. Of the 54 candidate loci with sufficient evidence of being under selection among both libraries, 28–39 were Arlequin–BayeScan *F*_st_ outliers. For LFMM, 45 candidates were associated with climate (of 54), 30 were associated with soil properties, and four were associated with plant health. Reanalysis of combined libraries showed that 42 candidate loci still showed evidence of being under selection. We conclude environment‐driven selection on specific loci has resulted in local adaptation in response to potassium deficiencies, temperature, precipitation, and (to a marginal extent) disease. High allele turnover along ecological gradients further supports the adaptive significance of loci speculated to be under selection.

## 1 Introduction

Understanding ecological pressures and their evolutionary impacts on natural tree populations represents an active research field in evolutionary ecology and is important to conservation of forests. There is little debate abiotic and biotic stressors can result in local adaptation and lead to evolutionary divergence of populations via isolation by adaptation (IBA) (Nosil, Funk, & Ortiz‐Barrientos, 2009). Local adaptation occurs widely in plants and animals, but the genetic basis is generally poorly understood (Fraser, Weir, Bernatchez, Hansen, & Taylor, 2011; Hereford, 2009; Leimu & Fischer, 2008). Studying the genetic basis of local adaptation, ecological factors driving divergent selection, and genetic differentiation of natural populations provides insights into how species may respond to future environmental changes, such as exotic pathogens, increasing deforestation, and future climate change (Fisichelli, Abella, Peters, & Krist, 2014). Answers to these questions are clearly relevant to conservation management of forest tree species.

We explore how environmental differences have influenced and will continue to drive evolution of natural populations of flowering dogwood trees (*Cornus florida* L.) using a population landscape genomic approach with genotyping‐by‐sequencing (GBS) data. *Cornus florida* is threatened by fungal pathogens, especially by powdery mildew (Li, Mmbaga, Windham, Windham, & Trigiano, 2009; Mmbaga, Klopfenstein, Kim, & Mmbaga, 2004; Windham, Trigiano, & Windham, 2005) and dogwood anthracnose (Redlin, 1991; Trigiano, Caetano‐Anollés, Bassam, & Windham, 1995; Daughtrey, Hibben, Britton, Windham, & Redlin, 1996; Zhang and Blackwell (2002); Holzmueller, Jose, Jenkins, Camp, & Long, 2006). The species is also subjected to abiotic environmental heterogeneities, such as variation in soil–nutrient composition and moisture, precipitation, temperature, different length of growing season, and exposure to sunlight, across its natural distributional range in eastern North America (Chellemi, Britton, & Swank, 1992; Holzmueller, Jose, & Jenkins, 2007; Kost & Boerner, 1985; Townsend, 1984). These variables may have resulted in local adaptation, for example, varying in flowering time from the coast to mountain regions (USA National Phenology Network). Additional background on the species is described in Supporting Information (Flowering Dogwood Background).

Population genomic and landscape ecology approaches (Anderson, Willis, & Mitchell‐Olds, 2011; Sork et al., 2013) provide means to detect local adaptation and loci responding to ecological forces of selection. Local adaptation can be revealed by genetic differentiation among populations at *F*_st_ outlier loci from contrasting environments as well as genetic correlation with environmental variables (Savolainen, Lascoux, & Merilä, 2013). The application of genomewide genetic markers (produced from next‐generation sequencing) to identification of truly adaptive loci still poses many challenges as a result of missing data from sequencing bias or sampling error. While limitations of analytical frameworks have been addressed using simulated data and through comparisons of methods (Lotterhos & Whitlock, 2014, 2015; Mita et al., 2013; Narum & Hess, 2011), bias of data resulting from next‐generation sequencing has remained a serious concern for marker‐based genomic approaches such as the recent but widely adopted RAD‐seq and genotype‐by‐sequencing (GBS) methods. Biases from such methods can contribute to frequent misidentification of false‐positive loci.

GBS and RAD‐seq methods are cost‐effective for sequencing a reduced genome sample from a large number of individuals, and they are noted for employing restriction enzyme digested libraries (RRL) that contain DNA fragments of specific target sizes to uncover loci with single nucleotide polymorphisms (SNPs) (Davey et al., 2011; Narum, Buerkle, Davey, Miller, & Hohenlohe, 2013). Both have been increasingly used for genetic mapping, population genomics, phylogeography, and phylogenetics (Baird et al., 2008; Davey & Blaxter, 2010; Eaton, 2014; Eaton & Ree, 2013; Gagnaire, Pavey, Normandeau, & Bernatchez, 2013; Hohenlohe et al., 2010; Lu et al., 2013; Qi et al., 2015; Recknagel, Elmer, & Meyer, 2013; Rubin, Ree, & Moreau, 2012). Application of GBS has demonstrated more powerful discernment of population genetic structure compared to microsatellite data and identification of more loci possibly responding to selective forces (Allendorf, Hohenlohe, & Luikart, 2010; Chu, Kaluziak, Trussell, & Vollmer, 2014; Gompert et al., 2014). While analysis of reduced genomes using this method is promising for identifying loci under selection, biases introduced by sequencing require cautious treatment of data in order to minimize false positives. Prior simulated studies have demonstrated failure to account for biases of reduced genome sequencing may result in both type I and II errors for detecting loci under selection (Davey et al., 2013). In particular, missing data and low coverage of SNP markers may erroneously characterize allelic variants as highly differentiated among populations, and even highly differentiated loci (measured by *F*_st_) may not have true adaptive value (Savolainen et al., 2013). Therefore, while the capability of genotyping large amounts of SNPs under possible selection has advanced, purging false positives from hundreds or thousands of candidate loci remains a bottleneck that hampers efficient exploration of true candidate genes. One approach to minimize false positive is to compare results from repeated and independent GBS experiments, but this approach has not been widely adopted due to added cost and labor involved.

In this study, we addressed the major concerns of the GBS method (specifically, repeatability and false positives due to missing data) using a combination of methods to more reliably identify loci under selection. First, we incorporated replication of sampling design into our sequencing strategy. Second, we isolated candidate loci that were detected by two *F*_st_ outlier‐based methods (Excoffier, Hofer, & Foll, 2009; Foll & Gaggiotti, 2008) and a genotype–environment association method (Frichot, Schoville, Bouchard, & François, 2013; Schoville et al., 2012) before reanalyzing them in a combined library with putatively neutral loci. For our final set of repeatedly genotyped loci showing evidence of local adaptation, we compared patterns of allele turnover along ecological gradients to our putatively neutral set of loci using a gradient forest (GF) approach recently applied to the field of ecological genomics (*Ellis, Smith, & Pitcher,* 2012; Fitzpatrick & Keller, 2015). Our main questions are as follows: (1) Has the species evolved local adaptation as a consequence of environmentally heterogeneous ecological pressures? (2) Which SNPs are likely to be candidates under selection? (3) Which environmental gradients are most important to genetic divergence and local adaptation of *C. florida* populations if any? (4) What genetic predisposition does *C. florida* possess to adapt to ongoing climate change in North Carolina? (5) And how does repeated GBS experimentation influence final results? The latter question is of utmost importance to researchers incrementally expanding sequencing‐based investigations across increasing portions of a taxon's range, and as such, we primarily report findings within North Carolina as part of a broader effort to characterize adaptive variation throughout the flowering dogwood range.

## 2 Materials and Methods

### 2.1 Site selection

**Figure 1:**
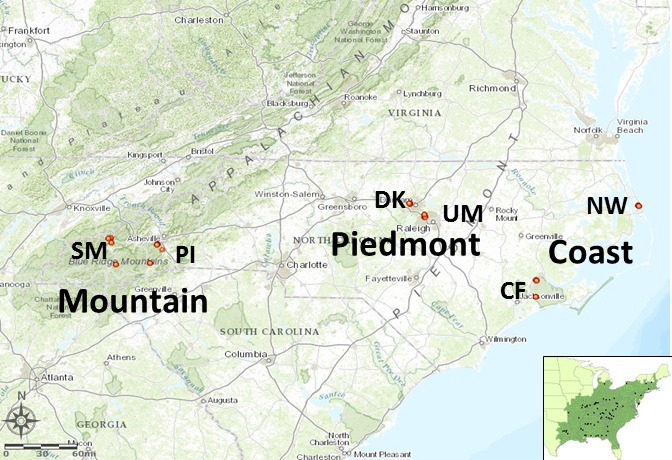
Map of sampling locations across North Carolina coast, Piedmont, and mountain regions—including the Great Smoky Mountains (SM), Pisgah Forest (PI), Duke Forest (DK), Umstead State Park (UM), Croatan Forest, and Nags Head Woods Ecological Preserve (NW). Bottom right inset represents entire range of *Cornus florida subsp. florida* sampled for broader range study

The natural range of *C. florida* comprises distinct and heterogeneous environments—spanning as far as north as Maine and occurring as a disjunct subspecies along the *Sierra Madre Oriental*; as such, various biotic and abiotic stressors have varied effects on the species in different ecoregions. Although ongoing research is underway to capture the full range of adaptive variation in *C. florida*, North Carolina is well suited for initial study as it encompasses three ecoregions with distinct environments spanning a range of longitudinal–elevational gradient similar to conditions of northern and southern portions of the species range (Wells, 1932). Therefore, we selected six populations within North Carolina, USA, representing divergent habitats and environments (Figure 1). These sampling areas represented mountains from within and around the Great Smoky Mountains National Park (GSMNP/SM) and Pisgah National Forest (PI), the Piedmont from Duke Forest (DK) and Umstead State Park (UM), and the Coastal region from Croatan National Forest (CF) and the Nature Conservancy site of Nags Head Woods Preserve (TNC/NW). These sites occurred along similar latitudes and represented the three distinct ecological regions of North Carolina (Figure 1, Table 1, Figure S1). Sampling sites were selected with consideration of their remoteness from developed areas to minimize the probability of studying cultivated trees. Due to high heterogeneity in elevation at small distances within mountainous regions, two mountain populations were each subdivided into two sampling sites. Two mountain locations for sampling were within national park and forest boundaries. Two other mountain locations were in close proximity to protected areas and were previously monitored for dogwood anthracnose disease by the NC Forest Service‐Forest Health Branch (Table 1; Figure S2). As the North Carolina Piedmont has been substantially developed, we chose two natural and relatively undeveloped locations (DK and UM). Our locations for sampling along North Carolina's coast were limited to upland mesic forests because flowering dogwoods rarely occur in the pocosin and other wetland communities of the mainland coast and outer banks. Environmental similarities of sites within ecological regions and differences of sites between ecological regions were confirmed by environmental data.

**Table 1:** Location and population summary statistics of sampled subpopulations within each ecological region of North Carolina

Subpopulation	Region	GPS coordinates	Sample	*H*_o_	*H*_e_	Nucleotide diversity
Library 1 dataset 82,697,746 paired reads 157,087 unfiltered loci 2,983 filtered loci 30.03× coverage						
Great Smoky Mountains	Mountains	35.57, −83.34	15	0.2591	0.2795	0.2899
		35.56, −83.31				
		35.51, −83.30				
Pisgah Forest	Mountains	35.49, −82.63	16	0.2524	0.2761	0.286
Umstead State Park	Piedmont	35.84, −78.76	16 (−2)	0.2704	0.28	0.2908
		35.87, −78.76				
Duke Forest	Piedmont	36.00, −78.97	19	0.2399	0.2547	0.262
Croatan Forest	Coastal Plains	35.03, −77.14	15	0.2652	0.2839	0.2944
		34.82, −77.15				
Nags Head Woods	Coastal Plains	35.99, −75.67	15	0.2838	0.2907	0.3013
Library 2 dataset 99,062,919 paired reads 151,271 unfiltered loci 2,764 filtered loci 34.57× coverage						
Great Smoky Mountains	Mountains	35.24, −83.24	15	0.278	0.2872	0.2976
Pisgah Forest	Mountains	35.25, −82.74	15	0.2529	0.2821	0.2926
Umstead State Park	Piedmont	35.84, −78.76	13	0.2745	0.2901	0.3021
		35.87, −78.76				
Duke Forest	Piedmont	36.00, −78.97	11	0.235	0.2673	0.2814
Croatan Forest	Coastal Plains	35.03, −77.14	15	0.2494	0.2863	0.2971
		34.82, −77.15				
Nags Head Woods	Coastal Plains	35.99, −75.67	15	0.2859	0.283	0.2932

### 2.2 Environmental variables

Three ecological regions from which natural populations were sampled are known to differ in temperature, rainfall, soil type, and disease incidence. Differences between mountain, Piedmont, and Coastal Plains regions of North Carolina were recorded with field‐site measurements. Environmental variables from each region were represented by data collected from two subpopulations, and each subpopulation consisted of one or two sites of 30 or 15 individual trees, respectively. Field measurements and soil cores were obtained in close proximity to each tree sampled, and the majority of sampled trees were spaced at least five meters apart within each subpopulation. With genotype evidence later obtained (see Genotyping and Data processing), relatedness between individuals was checked using PLINK (Purcell et al., 2007) to ensure environmental data affiliated with clonal or sibling pairs were excluded.

Environmental measurements included elevation, proximity to water, canopy coverage, and 15 soil core features (Table S1 Appendix) and were recorded at sites during sample collection (described in Environmental Variables, Supporting Information). Additional environmental data (soil classification, temperature, precipitation, frost‐free period, and length of growing season) were obtained via GIS (see GIS Resources, Supporting Information). We note further in Supporting Information that the size of our environmental dataset was reduced for certain analyses, namely GF analysis. As collinearity among variables and its effect on the random forest algorithms (that GF is an extension of) are not fully understood, we safeguarded against any possible problems by reducing the number of collinear pairs of environmental variables. Prior to reduction of collinearity for GF (Additional Validation of Environmental and SNP Data, Supporting Information), our environmental dataset consisted of 12 variables (Table S1 Appendix), excluding 15 soil core measurements and soil types from the USGS soil classification scheme.

### 2.3 Functional traits

Two functional plant traits, plant health and leaf osmotic potential, were measured in this study. Plant health was measured during plant collection. We measured the health condition of every sampled tree using a visual estimation method (Mielke & Langdon, 1986) employed previously by forest health monitors. Individuals were scored for one of five categories based on twenty percentile increments of tree canopy displaying symptoms of disease infection (e.g., leaf blotting, necrosis, or branch dieback). Individuals rated with a score of five exhibited minimal or no stress (0%–20% canopy infection), while individuals with scores of one had almost no living or disease‐free foliage (80%–100% canopy infection). In addition, we employed an alternative binary scoring system that recorded scores of four and five as one and anything below as a score of zero. After assigning each tree a health score, at least four branch cuttings were taken from the majority of sampled trees (except some mountain trees with substantial branch dieback) and transported to the laboratory for leaf osmotic potential measurements using an osmometer.

We designed osmometer experiments to specifically measure leaf osmotic potential (tendency of water to move into and be retained in mesophyll cells), which is indicative of plant drought tolerance (Bartlett, Scoffoni, & Sack, 2012). Branches were randomly selected by cutting from each sampled tree. Cuttings were placed immediately in 50‐ml vials filled with water and transported promptly to a common room temperature‐controlled setting where measurements were taken using an osmometer (described in Functional Traits, Supporting Information).

### 2.4 Genotyping

Fresh leaf samples were collected from the same plants visually scored for health in the field. Samples were stored at −20°C until they were used for DNA extraction. A total of 180 trees were sampled from six populations, with 30 samples from each (Table 1). These samples were divided into two sets, and each set contained approximately half of the samples from each subpopulation. A GBS library was prepared for each of the two sets (96 and 85 individuals) for sequencing with Illumina HiSeq 2000. DNeasy Plant Mini kits (Qiagen, Inc., Valencia, CA, USA) were used to extract DNA from frozen fresh leaf tissue. Quantity and quality of extracted DNA were checked using fluorescent dye‐binding (PicoGreen) assays, agarose gels, and UV absorbance (Nanodrop). DNA samples with poor quality were purified with Qiagen DNA Purification kits or re‐extracted until good‐quality DNA (A_260_/A_280_>1.7) was available. GBS libraries were prepared for two DNA libraries of 96 and 85 individuals separately, according to the double‐digest RAD‐seq/GBS method (Peterson, Weber, Kay, Fisher, & Hoekstra, 2012; Supporting Information). The two libraries with different pooled individuals were sequenced on two different flow cells on an Illumina Hiseq.

### 2.5 Data processing

Sequence data from each Illumina library were cleaned by removing contaminant and low‐quality sequences. High‐quality reads from each library were independently assembled de novo and filtered again after assembly. Paired‐end one (PE1) reads were processed separately for each of the two libraries (see discussion of PE2 reads, Supporting Information). GBS barcode splitter, other custom Perl scripts, and FASTX‐Toolkit were used to sort samples by barcode, trim PE1 reads to 90 bps, and remove sequence reads that had more than 5% of their bases with a quality score below 20. Bowtie 2 (Langmead & Salzberg, 2012) was used to align raw reads to several fungal genomes in order to identify and filter out as many contaminant DNA fragments as possible. Following these steps, we processed sequences into catalogs of shared loci using STACKS (Catchen, Amores, Hohenlohe, Cresko, & Postlethwait, 2011; Catchen, Hohenlohe, Bassham, Amores, & Cresko, 2013).

After removing nontarget sequences, remaining sequences were processed through STACKS version 1.19 (Catchen et al., 2011) in order to assemble sequences de novo into two libraries of shared reads (or 90 bp RAD‐tag loci with one to four SNPs per RAD‐tag). The following parameter options for ustacks, cstacks, and sstacks were specified as *m* 3 [minimum coverage to create stack], *M* 2 [maximum nucleotide distance permitted between initial stacks], *N* 4 [maximum nucleotide distance permitted between secondary stacks], *max locus stacks* 3 [maximum number of stacks to consider an assembled locus], and *n* 2 [mismatches allowed between tags from different samples]. In addition to this parameterization (justified in SNP Data processing, Supporting Information), we also chose filtering parameters that controlled the amount of missing data tolerated for population genetic analyses.

Missing data were also important factors to consider for processing steps. A common practice is to use >20% missing data criterion as an arbitrary cutoff to exclude loci in datasets (Narum et al., 2013), but some have relaxed the criterion to up to 80% missing data (Crossa et al., 2013). Excessive data filtration can have unforeseen consequences (Huang & Knowles, 2014) due to truncation of loci with higher mutation rates and reducing statistical power of analyses. We relaxed our missing data acceptance threshold slightly by keeping loci with a maximum of 25% missing data in each library's samples. We also designated a 5% minor allele frequency cutoff to reduce artifacts of sequence and assembly error. After extensive exploratory tests of fundamental filtering parameters and inspection of preliminary results with PCA (implemented in the R package adegenet, Jombart, 2008), we removed two individuals from the first library of 96 samples due to suspicions of being clonal pairs of a planted cultivar. One individual from the second library of 85 individuals was removed due to considerable amounts of missing data, likely a result of failure to amplify sequence fragments during sequencing. Data with these crucial adjustments were used for further analyses to infer population genetic structure and identify candidate loci under selection, and additional adjustments and SNP validation were conducted depending on the type of analysis (Additional Validation of Environmental and SNP Data*,* Supporting Information).

### 2.6 Identification of candidate loci under selection

To identify loci strongly deviated from the general population genetic structure and strongly associated with environmental differences, we first characterized individuals’ membership to biological clusters. Using a dataset of uncorrelated SNPs not in linkage disequilibrium for our two libraries (first occurring SNP per RAD‐tag), STRUCTURE (Pritchard, Stephens, & Donnelly, 2000) was implemented for the first eight cluster models (*K* = 1–8) using ten replicate analyses each with a burn‐in of 100,000 and 100,000 subsequent iterations. The same procedure was carried out on the combined library of 1,171 putatively neutral SNPs in Hardy–Weinberg equilibrium (Additional Validation of Environmental and SNP Data, Supporting Information).

We then scanned for outlier loci deviating from the simulated null distribution of heterozygosity *F*_st_ for hierarchically structured populations using the method of Excoffier et al., 2009 (implemented in Arlequin; Excoffier & Lischer, 2010) on the highest *F*_st_ SNP for each RAD‐tag. A coastal‐mainland hierarchical population structure, identified as the best grouping from STRUCTURE, AMOVA, and PCA analyses, was designated for *F*_st_ outlier loci analysis using Arlequin. Using Arlequin, we ran 20,000 simulations with 10 simulated groups and 100 demes per group in the analysis to identify candidate loci under selection. Using another extension of the *F*_st_ outlier approach (Beaumont & Nichols, 1996) implemented in BayeScan (Foll & Gaggiotti, 2008), we also assessed allele frequencies from the same datasets to test whether loci were highly differentiated when parameterizing a classical island model instead of a hierarchical island model. Under certain simulated scenarios where adaptive variation conflicted with a defined hierarchical neutral structure, BayeScan has been shown to outperform Arlequin (Narum & Hess, 2011), and other simulated work suggests comparison of results from different outlier methods can reduce error rates (Villemereuil, Frichot, Bazin, François, & Gaggiotti, 2014). The analysis was performed with the following priors: 5,000 sample size; 20 thinning intervals; 20 pilot runs of length 5,000; 100,000 additional burn‐in; uniform distribution between 0 and 1; and a prior odds for neutrality of 5:1. Prior odds of 1:1 and 10:1 for the neutral model were also evaluated in BayeScan.

Significant genotype–environment association (GEA) was investigated using latent factor mixed modeling (LFMM; Frichot et al., 2013). LFMM accounts for covariation of alleles and environment, and compared to other GEA tests, more flexibly accounts for hidden population structure while maintaining a relatively lower false detection rate under models of hierarchically structured populations (Villemereuil et al., 2014). As we found evidence to support subpopulations being hierarchically nested within two larger clusters (coastal‐mainland), we chose LFMM to identify candidate loci. The optimal latent factor number (*K* = 2), identified with the Evanno method for STRUCTURE analysis (Evanno, Regnaut, & Goudet, 2005), was incorporated in LFMM. For LFMM, we ran the analysis with 50,000 sweeps for each pairwise test with a burn‐in of 12,500 sweeps because repeated tests of parameters showed a precise consensus in regard to SNPs being detected as highly associated with environmental and functional traits.

*F*_st_ outliers from Arlequin analysis were filtered for *p*‐values below 5%, and a *q*‐value for each locus was subsequently calculated by the program QVALUE (Benjamini & Hochberg, 1995) to monitor false discovery rates of positive results. Q‐values of outlier loci from BayeScan were automatically calculated by the program, and those results were filtered to retain loci with a *q*‐value below 0.1. Results from LFMM genotype–environment associations were filtered to keep significant associations with a *Z* score over 4, following the practice of Frichot et al. (2013). The score corresponded to a Bonferroni alpha correction of 0.01 for 1,000 SNPs.

### 2.7 Detecting allele turnover patterns along ecological gradients: gradient forest and mantel tests

A small subset of loci (54 RAD‐tags) had compelling evidence for being under selection, defined as being detected by multiple methods across libraries (three or more overlaps in Figure 6a) and consistently genotyped across libraries, were selected for analysis of allele turnover along ecological gradients using gradient forests analysis. This analysis is a novel application of a community ecology method (*Ellis* et al.*,* 2012) to study ecological genomics of local adaptation. The method was recently demonstrated by Fitzpatrick and Keller (2015) to be useful for further evaluating the adaptive and ecological significance of putative candidate loci under selection and for determining the relative importance of various ecological pressures on the adaptive landscape. A larger set of presumably neutral loci (1,307 RAD‐tags) were constructed as the reference group for the analysis. The “reference loci” were consistently genotyped across libraries but were not identified as candidates under selection in any of the Arlequin, BayeScan, and LFMM analyses. To distinguish departures of candidate SNPs from the general genomic background, we concurrently analyzed and plotted patterns of allele turnover along ecological gradients for the both the candidate and reference subsets of our dataset using GF analyses (Fitzpatrick & Keller, 2015). The 176 individual trees were treated as response variables for GF. On the other hand, the subpopulations (two mountain populations subdivided) were considered for pairwise matrices used in mantel tests. Mantel tests were applied to the same datasets to corroborate overall correlations (instead of SNP‐specific patterns) between environment and candidate‐reference loci, after controlling for geographic distance (Legendre & Fortin, 1989). Mantel tests, specifically partial mantel tests, have been similarly applied in recent population‐level studies (Zhao et al. 2013). Before implementing GF and mantel procedures, we implemented one further series of validation procedures to our environmental data, candidate loci, and reference loci as described in Supporting Information (Additional Validation of Environmental and SNP Data).

GF analyses were conducted with the gradientForest R package (Smith & Ellis, 2013), using only SNPs with a variable correlation threshold of 0.5 or greater to generate plots of allele turnover. As a precaution, we minimized the nonindependence of SNPs in our genetic dataset prior to GF analysis because (although not demonstrated to affect GF specifically) linkage disequilibrium was known to bias landscape and population genomic approaches by adding weight of inference to correlated loci pairs. To reduce GF's susceptibility to linkage disequilibrium, only one SNP per RAD‐tag was considered while fitting the GF model using 2,000 regression trees. A random SNP per RAD‐tag was selected for reference loci, but the SNP with the highest *F*_st_ per RAD‐tag was chosen for candidate loci. SNP data were converted to presence–absence of the minor allele for each of 176 individuals (two samples duplicated among two libraries) and were analyzed in GF using the regression model, which was a standard implementation of the gradientForest R package. Remaining parameters to fit GF models were selected according to Fitzpatrick and Keller (2015).

Partial mantel tests were performed with R ade4 and ecodist packages (Chessel, Dufour, & Thioulouse, 2004; Goslee & Urban, 2007) using Slatkin's linearized *F*_st_ data to ensure genetic patterns were suited for linear regression. Pairwise matrices of linearized *F*_st_ values were obtained from Arlequin, and for every environmental–functional variable, each subpopulation's mean was calculated. The pairwise difference between subpopulations’ means was then determined to obtain a dissimilarity matrix for each environmental–functional trait. Geographic distances between populations were calculated using Euclidean distances derived from a projected coordinate system (in meters) to provide control for isolation by distance while detecting the significant correlations between overall genetic and environmental distances (i.e., partial mantel tests). Full and partial mantel tests were carried out independently for each environmental–functional trait.

## 3 Results

### 3.1 Environmental and functional trait differences

Results of one‐way ANOVA (or Kruskal–Wallis tests for environmental data not fitting ANOVA assumptions) indicated the majority of environmental features were significantly different (*p* < .05) between at least two of six populations compared (Table S1 Appendix). Several soil features, however, did not show significant differences among sampled locations (e.g., Ca, Mg, Cu, Zn, CEC, exchangeable acidity, pH, and base saturation). Tukey–Kramer tests (or Dunnett's modified Tukey–Kramer tests for violations of homoskedasticity) supported observations that environmental features vary along the gradient of mountain, Piedmont, and coastal regions (Table S1 Appendix). Higher elevation populations were wetter per month and shorter in growing period compared to those of populations at lower elevations (Table S1 Appendix). Humidity in Piedmont locations was slightly lower than in coastal locations. Coastal populations were environmentally differentiated from other populations in soil features, especially for density (mg/dm^3^) of sodium, potassium, manganese, and soil weight to volume ratios (Table S1 Appendix).

Mean values of plant health rating varied significantly among populations (Figure S2), but leaf osmotic potential varied to a lesser extent (Table S1 Appendix). Results of Tukey–Kramer tests confirmed mountain populations had significantly lower plant health ratings than other populations (Mountain: 3.33, 3.03; Piedmont: 4.67, 4.48; Coastal: 4.77, 4.77; Table S1 Appendix). In contrast, population differences of leaf osmotic potential were relatively modest, showing a west–east gradient of osmolality (mmol/kg) from lower values in mountain populations to higher values in coastal populations.

### 3.2 Genotyping results

Before removing contaminant reads aligned to fungal genomes and low‐quality sequences, libraries one and two had a total of 82,697,746 and 99,062,919 paired sequences, with an average of approximately 861,435 and 1,179,320 PE1 sequences per individual, respectively (Table 1). After filtering sequences with low‐quality scores (sequence reads with >5% of bases below a quality score of 20) prior to assembly with STACKS, 777,928.2 and 1,005,628 90 bp PE1 sequences per individual (on average) remained for library one and two. After de novo assembly of sequence reads using STACKS, the average coverage of reads per assembled stack for a given individual in the two libraries was 30.03× and 34.57×, respectively, and a total of 157,087 and 151,271 unique SNPS were recovered (Table 1). After removal of rare SNPs (minor allele frequencies <5%) and loci missing in >25% of individuals per library, the number of unique SNPs was reduced to 2,983 and 2,764 for library one and two, respectively. When only a single SNP per locus‐tag was retained, numbers were further reduced to 2,170 and 1,994. When both libraries’ results were examined together, a total of 2,533 unique loci were identified (Figure S4). Of these unique SNPs, a total of 1,631 loci were repeatedly genotyped in both libraries (Figure S4A)—representing approximately 75% and 82% of the total of each library.

### 3.3 Population genetics

**Figure 2:**
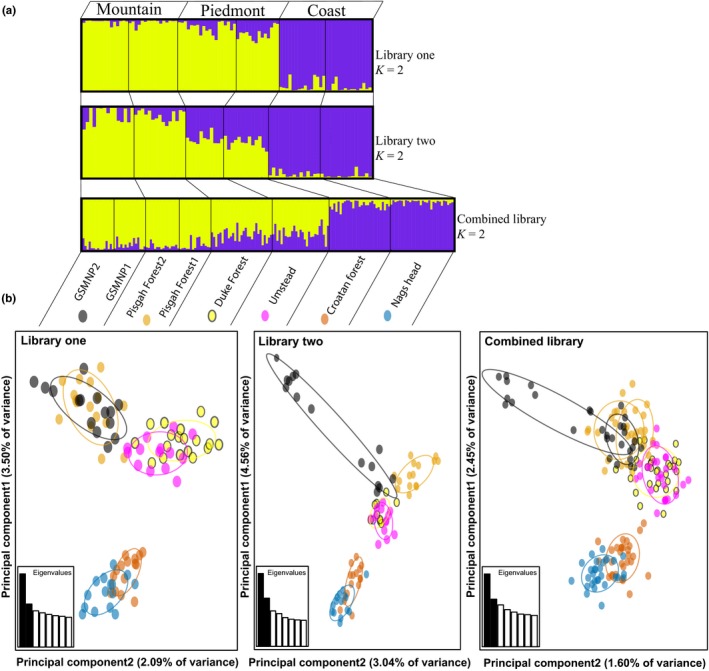
Analyses of overall genetic population structure for library one, library two, and combined datasets including (a) STRUCTURE results of latent factor *K* = 2 model and (b) principal component analysis of 94 and 84 individuals from each dataset. Two individuals removed because of possible hybridization with planted cultivar tree in library one, and one individual removed in library two because of insufficient amplification and sequencing of genotypes. Prior to analysis of each library, only first occurring SNP per RAD‐tag was considered in order to reduce linkage disequilibrium. Only 1,171 reference SNPs (validated by selection tests to be putatively neutral) in Hardy–Weinberg equilibrium for more than four subpopulations were used to analyze population structure of combined library

STRUCTURE analyses of both libraries supported an optimal *K* = 2 grouping of individuals, a coastal population group and a mainland (mountain‐Piedmont) group (Figures 2a and S5). UPGMA dendrograms of genetic distances (Nei, 1972) generated with the program Populations (Langella, 1999) also showed high support for a grouping of coastal subpopulations that was distinct from mainland populations (Figure S6). PCA of both library one and two data similarly showed two distinct clusters defining a mountain‐Piedmont group and a coastal group (Figure 2b). One mountain subpopulation (GSMNP) in library two showed additional intrapopulation clustering. Overall, these results clearly indicate at least two genetic clusters—distinguishing coastal populations from mainland populations.

AMOVA results (Table 2) showed a considerable percentage of total genetic variation attributable to differences among individuals (library one: 92.72%, library two: 88.27%) and a small but significant percentage was attributed to differences among subpopulations within a two group hierarchical structure (library one: 1.93%, FSC = 0.01993, *p* < .001; library two: 3.34%, FSC = 0.03448, *p* < .001). Differences between coastal and mountain‐Piedmont groups accounted for approximately 3% of total genetic variation for both library one and two results, which is marginally insignificant with a two‐tailed statistical test (library one: *p* = .06585; library two: *p* = .06707). This suggests extensive genetic mixture within regions and frequent gene flow or weak genetic differentiation between coastal and mainland populations. STRUCTURE and AMOVA results from analyses for a less probable hierarchical population structure of mountain, Piedmont, and coast division are available in Supporting Information (Table S2; Figure S8).

**Table 2:** AMOVA results from separate analyses of library one and two datasets. Two groups represented are the coastal group and the mainland group (mountains and Piedmont). 50,000 permutations ran in Arlequin for test of significance

	Variation source	*df*	Percentage of variation	*p*‐Value
Library one dataset	Among groups	1	3.04	.06585
	Among populations within groups	4	1.93	<.001
	Among individuals within populations	88	2.31	.08434
	Within individuals	94	92.72	<.001
Library two dataset	Among groups	1	3	.06707
	Among populations within groups	4	3.34	<.001
	Among individuals within populations	78	5.38	.00134
	Within individuals	84	88.27	<.001

### 3.4 Identification of candidates under selection

**Figure 3:**
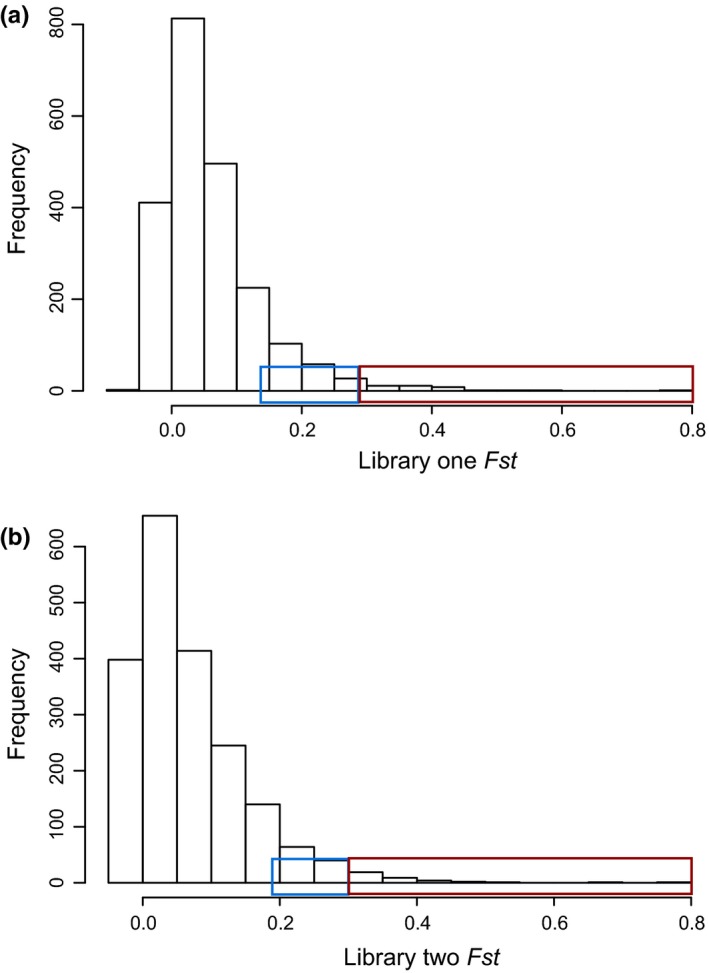
Number of SNPs with variable degrees of population genetic differentiation. *F*_st_ distributions of highest *F*_st_ SNP per RAD‐tag loci passing filtering criteria for library one and two. *F*_st_ values estimated in Arlequin using a mainland coastal hierarchical structure. Range of *F*_st_ outlier SNPs in ninety‐five percentile boxed in blue and *F*_st_ outlier SNPs in ninety‐ninth percentile boxed in red

The distribution of SNPs’ *F*_st_ values estimated by Arlequin from a coastal‐mainland hierarchical structure shows a majority of loci have a *F*_st_ value below 0.25, and a very small portion have a *F*_st_ value of 0.25–0.8 corresponding closely to the ninety‐ninth significance percentile (Figures 3 and S7). Analysis with Arlequin revealed 151 and 216 outlier loci beyond the 5% or 1% *p*‐value level for libraries one and two, respectively (Figures 6 and S7, *F*_st_ and *q*‐values in Table S1). Among these loci, 54 were consistently detected in both libraries. Analyses using BayeScan found 43 and 37 outlier loci from library one and two, respectively, passing a *q*‐value cutoff of 0.1. Two loci identified as *F*_st_ outliers in BayeScan results were common to both libraries and also matched significant Arlequin results from each library (Figure 6a, 1+1 in bottom right quadrant).

**Figure 4:**
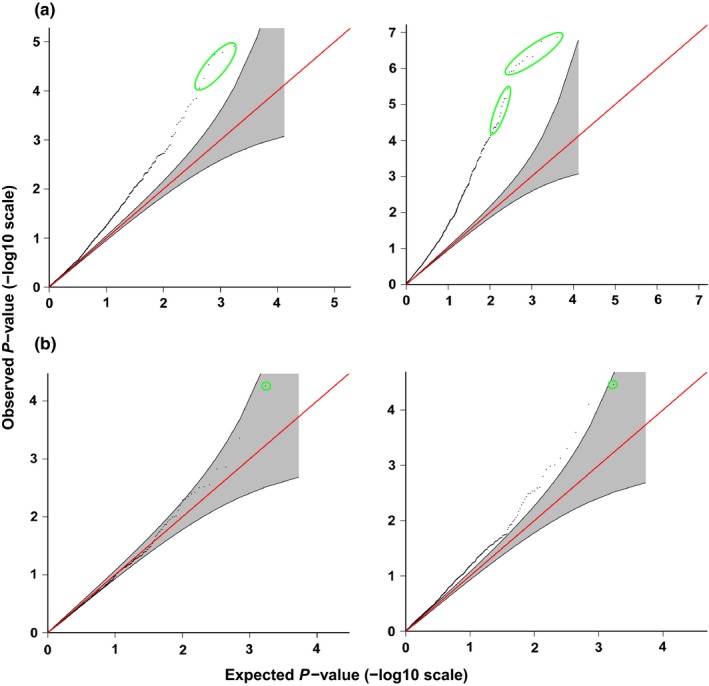
Select Q‐Q plots from LFMM (*K* = 2) analyses of genotype–environment associations—including visualizations of associations to top two principle components of environmental distances for samples in (a) library one and (b) combined library. Dots in green ovals indicate significantly associated SNP markers with a *Z* score >4). Reduction of false positives is considerably reduced in combined library (part B), which consists of 1,171 putatively neutral SNPs and 43 candidate loci

**Figure 5:**
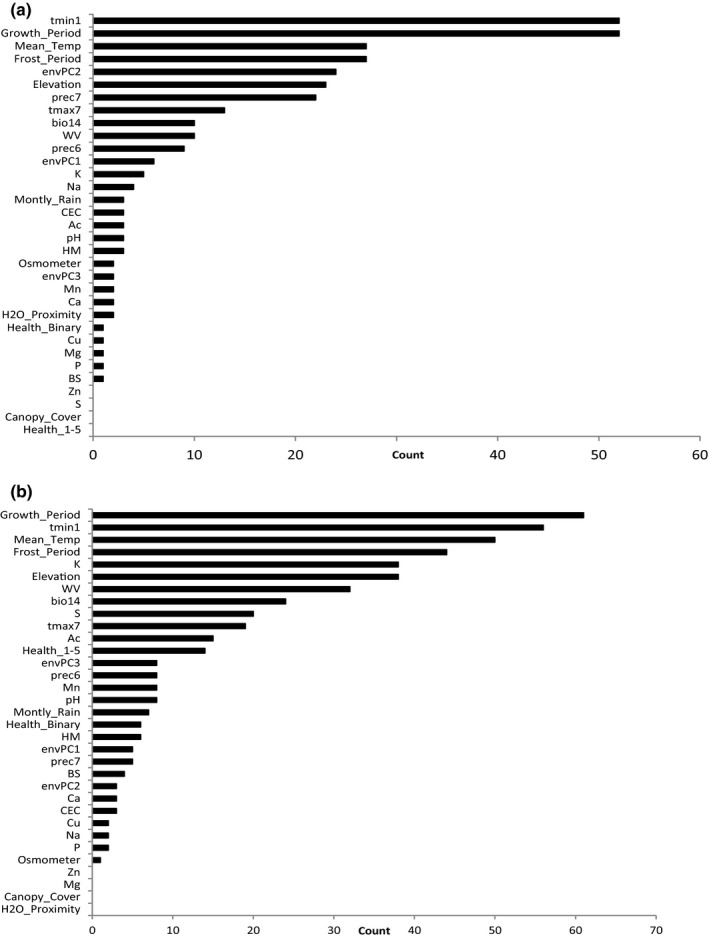
Total count of loci (*x*‐axis) with SNPs passing *Z* cutoff of 4 for association to given environmental variables (*y*‐axis) using LFMM (*K* = 2) analysis of (a) library one and (b) library two

**Figure 6:**
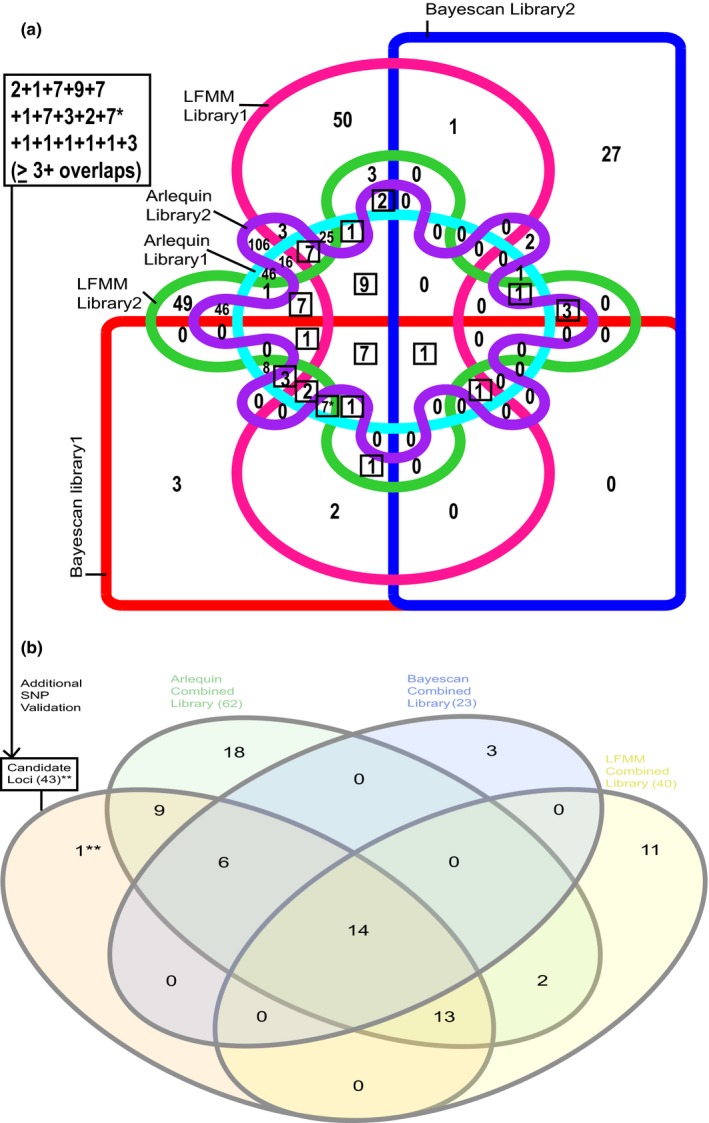
Venn diagrams comparing: (a) total candidate loci detected by LFMM, Arlequin, and BayeScan in libraries one and two and (b) in combined library of 1,171 putatively neutral SNPs and 43 candidate loci. After additional validation of SNPs (Additional Validation of Environmental and SNP Data, Supporting Information), 43** of 54 candidate loci consistently genotyped across libraries and detected to be under selection by at least three tests of local adaptation (boxed in Figure 6a) were reanalyzed by selection tests with 1,171 putatively neutral loci (no overlaps in part a). One overlap in part A originally represented 13 loci detected by all three selection tests in library one but was reduced to seven results (7*) because six loci were not genotyped successfully in library two

Results from single locus tests implemented by LFMM indicated 129 (library one) and 133 (library two) RAD‐tags had at least one SNP correlated (*Z* scores > 4) with one or more of the following: 12 environmental variables, 15 soil properties, leaf osmotic potential, two scales of plant health, and three reduced principal components of environmental distances. *Z* scores of 4 correspond to *p*‐values ≤ .01 with a Bonferroni correction for 1,000 loci. Q‐Q plots from LFMM (Figure 4) showed that reduced‐dimension environmental components derived from features such as soil moisture, pH, nutrients, length of growing period, mean annual temperature, and mean monthly rainfall (Figure S3) had an excess of significant genotype–environment associations when analyzed in a single library alone, but results were much more conservative when the combined library of candidate SNPs was reanalyzed with putatively neutral SNPs. LFMM association tests between each SNP and each individual environmental or functional trait revealed several significant correlations while controlling for hidden population genetic structure among individual trees (Table S1; Figure 5). Similar to results of GF and partial mantel tests, temperature covariates were most often correlated with outlier SNPs. LFMM analyses did not detect significant correlation between genetic data and canopy coverage or levels of zinc. Significant correlations of genetic data to functional traits detected by LFMM analyses differed between the two libraries. When coding plant health as a binary state, more significant associations with SNPs were detected in library one than in library two. However, when plant health was scored using a 1–5 scale, the pattern was reversed. Of loci detected to be putatively under selection according to Arlequin, BayeScan, and LFMM, a subset of 54 loci were detected in more than one of these methods and genotyped in both libraries (Figure 6a).

The rate of consistently genotyping a locus across libraries was 75%–82%, whereas the rate of consistently identifying SNPs as candidates under selection by at least one method ranged between 23%–32% when considering any genome scan analysis of library one and two. See Supporting Information (Defining Consistency) for our definition of consistency. A smaller subset of candidates under selection were identified as highly interesting as they were detected between both libraries by multiple *F*_st_ outlier and correlation tests, and several candidates matched to elements of *C. florida*'s transcriptome or the NCBI nonredundant (nr) sequence repository. The conservative subset of 54 SNPs had extensive evidence to support adaptive significance and was defined by having three or more overlaps from Figure 6a in addition to being present in both filtered datasets of library one and two. In other words, any three of the six criteria were met to consider the 54 consistently genotyped loci as candidates: (1) Arlequin‐library one significance; (2) Arlequin‐library two significance; (3) BayeScan‐library one significance; (4) BayeScan‐library two significance; and any significant environmental‐SNP associations for LFMM analysis of (5) library one and (6) library two.

For the 54 SNPs identified as candidates of selection in at least three analyses (those falling in the three overlapping areas boxed in Figure 6a) and consistently genotyped across libraries, BLAST searches to the NCBI nr repository showed eight loci with hits to predicted gene products (Table 3), and seven loci had no hits to predicted functions but aligned to the transcriptome of *C. florida* (Zhang et al., 2013). Evidence in support of these specific candidate SNPs is documented further in Table 3, and curated annotations with clear adaptive significance are examined further in discussion. Notable trends observed among the 54 candidate loci summarized on Table 3 are reported here. Of the 54 candidate SNPs, *F*_st_ estimates were consistently high among Arlequin analyses of both libraries for 39 of our 54 candidates. BayeScan estimates of *F*_st_ were consistently lower, but 28 of 54 candidate SNPs had been detected as an *F*_st_ outlier at least once by BayeScan analyses of the two libraries. According to LFMM results of the 54 candidate loci, 45 loci had at least one correlation to climatic data, 30 were significantly associated with at least one soil property, and four were correlated to visual health scores. Given the relatively high adaptive significance of these 54 candidates SNPs compared to the general pool of reference SNPs, we expected and found clear differences in regard to patterns of allele turnover along ecological gradients.

**Table 3:** List of 54 candidate loci under selection that meet the following criteria: consistently genotyped across libraries and repeatedly called as a candidate under selection in Arlequin, BayeScan, or LFMM (visualized by at least three overlaps in Figure 6a). Evidence in support of each accession includes positive results of *F*_st_ outlier and LFMM association tests as well as predictions of gene function and alignment to *Cornus florida* transcriptome contigs

Unifier ID	Library One ID	Library Two ID	Matching GenBank accession	Arlequin (Library1) significance (p, q, *F*_st_)	Arlequin (Library2) significance (p, q, *F*_st_)	BayeScan (Library1) significance (alpha, PP, *F*_st_)	BayeScan (Library2) significance (alpha, PP, *F*_st_)	Significant LFMM (Library1) environment function trait associations	Significant LFMM (Library2) environment function trait associations	Sequence description	Gene ontologies	Match to *Cornus florida* transcriptome
B332	2117	60071	XM_006493426	1.00E‐07, 0, 0.478658	0.0013301, 0.07104953, 0.313298	1.3431, 0.9898, 0.15911	Not significant	GROWING PERIOD, TMIN1, PREC6, PREC7, ENVPC2	Potassium, frost period, mean temp, growing period, tmin1	Probable glycerol‐3‐phosphate dehydrogenase	C:glycerol‐3‐phosphate dehydrogenase complex; F:glycerol‐3‐phosphate dehydrogenase [NAD+] activity; F:NAD binding; P:carbohydrate metabolic process; P:glycerol‐3‐phosphate catabolic process; P:oxidation–reduction process; P:glycerolipid metabolic process	Yes
B1350	9249	43811	XM_00648094	0.0086562, 0.1129688, 0.252691	Not significant	1.3013, 0.95839, 0.15552	Not significant	Elevation, copper, frost period, mean temp, growing period, tmin1, tmax7	No significant associations	Probable lrr receptor‐like serine threonine–protein kinase at3 g47570 isoform x1	C:integral component of membrane; F:protein serine/threonine kinase activity; F:ATP binding; P:protein phosphorylation; P:serine family amino acid metabolic process	Yes
B982	6702	8476	CP002687	0.0297114, 0.1843343, 0.201058	0.0240841, 0.1371417, 0.206488	1.0959, 0.79996, 0.14063	Not significant	No significant associations	No significant associations	L‐type lectin‐domain containing receptor kinase‐like (transcriptome contig: scaffold 19651)	F:carbohydrate binding	Yes
B1092	7448	59746	ACUP02003346	Not significant	0.00796913, 0.09401618, 0.258492	Not significant	1.5006, 0.9916, 0.20093	No significant associations	Weight/volume (soil), potassium, frost period, mean temp, growing period, tmin1	Serine threonine–protein phosphatase pp1‐like	F:phosphoprotein phosphatase activity; P:protein dephosphorylation	Yes
B1219	8439	64761	XM_010110700	1.00E‐07, 0, 0.52692	0.00772755, 0.09401618, 0.279965	1.6494, 0.9996, 0.19998	Not significant	Frost period, mean temp, growing period, tmin1, prec7, envPC2	No significant associations	Valine–tRNA ligase partial mRNA	F:nucleotide binding	Yes
B768	5106	39512	XM_002520327	0.0180767, 0.1527465, 0.243998	0.00238949, 0.07107841, 0.302617	1.1064, 0.86697, 0.1361	Not significant	Sodium, frost period	Potassium, growing period	ATP‐dependent zinc metalloprotease FTSH protein	C:integral component of membrane; F:metalloendopeptidase activity; F:ATP binding; F:zinc ion binding; P:proteolysis	Yes
B757	5042	78313	XM_011086966	1.00E‐07, 0, 0.752807	1.00E‐07, 3.32E‐05, 0.773273	2.0433, 1, 0.26411	Not significant	prec7, envPC2	Humic matter (soil), weight/volume (soil), potassium, sulfur, sodium, frost period, mean temp, growing period, tmin1	Chlorophyll a b binding protein	C:membrane; P:photosynthesis, light harvesting	Yes
B124	639	64462	ABC59094	0.014921, 0.1129688, 0.228029	0.0146964, 0.1200407, 0.225507	Not significant	Not significant	prec7	No significant associations	Cytochrome p450 704c1‐like (transcriptome contig: C350477)	F:monooxygenase activity; F:iron ion binding; F:oxidoreductase activity, acting on paired donors, with incorporation or reduction of molecular oxygen; F:heme binding; P:oxidation–reduction process	Yes
B1337	9193	59829	N/A	0.00296975, 0, 0.345834	0.000302531, 0.03510016, 0.479563	Not significant	Not significant	Growing period, tmin1, envpc2	Weight/volume (soil), growing period, tmin1	N/A	N/A	Yes
B634	4368	7819	N/A	0.00104285, 0, 0.422817	0.0142438, 0.1177921, 0.255863	0.9668, 0.85937, 0.11872	Not significant	Growing period, tmin1, prec7, envPC2	Growing period, tmin1	N/A	N/A	Yes
B705	4744	64731	N/A	0.0212011, 0.1527465, 0.230035	0.00502886, 0.08608937, 0.332181	Not significant	Not significant	tmin1	Humic matter (soil), weight/volume (soil), potassium, sulfur, mean temp, growing period, tmin1	N/A	N/A	Yes
B1217	8430	55186	N/A	0.0232869, 0.1527465, 0.210864	0.0281231, 0.1458679, 0.193064	0.92198, 0.78336, 0.11718	Not significant	No significant associations	Potassium, mean temp, growing period, tmin1	N/A	N/A	Yes
B947	6502	10273	N/A	0.0220645, 0.1527465, 0.236571	0.0133702, 0.1140064, 0.241524	Not significant	Not significant	Growing period, tmin1, envPC2	No significant associations	N/A	N/A	Yes
B1408	9769	81606	N/A	0.0234086, 0.1527465, 0.217577	0.000805608, 0.06026913, 0.436111	Not significant	Not significant	No significant associations	Elevation, health score (1–5), weight/volume (soil), potassium, frost period, mean temp, growing period, tmin1, bio14	N/A	N/A	Yes
B5	36	6203	N/A	Not significant	0.0297637, 0.1494183, 0.211722	Not significant	1.2511, 0.92318, 0.16907	No significant associations	Manganese	N/A	N/A	Yes
B1042	7171	3392	N/A	0.0127384, 0.1129688, 0.265077	0.0435917, 0.162696, 0.164033	Not significant	Not significant	Mean temp, growing period, tmin1	No significant associations	N/A	N/A	No
B1098	7510	63181	N/A	1.00E‐07, 0, 0.424367	1.00E‐07, 3.32E‐05, 0.521851	Not significant	Not significant	prec7	Sodium, tmin1	N/A	N/A	No
B1111	7644	45882	N/A	0.0224023, 0.1527465, 0.234922	0.0338638, 0.1532725, 0.184122	Not significant	Not significant	No significant associations	Health score (1–5), humic matter (soil), weight/volume (soil), exchangeable acidity (soil), sulfur, mean prec, health score (0–1)	N/A	N/A	No
B114	577	69202	N/A	0.0223518, 0.1527465, 0.21234	0.0338675, 0.1532725, 0.19121	Not significant	Not significant	prec7, envPC2	Potassium	N/A	N/A	No
B115	581	37454	N/A	0.0107988, 0.1129688, 0.284727	0.00939402, 0.096248, 0.296292	Not significant	Not significant	envPC2	Elevation, weight/volume (soil), potassium, sulfur, frost period, mean temp, growing period, tmin1, bio14	N/A	N/A	No
B1160	7998	85678	N/A	0.000390918, 0, 0.331331	Not significant	Not significant	Not significant	Growing period, tmin1, prec7	prec7, envPC2	N/A	N/A	No
B1189	8230	63202	N/A	0.010991, 0.1129688, 0.262808	0.0179029, 0.1252447, 0.21726	Not significant	Not significant	Growing period, tmin1	No significant associations	N/A	N/A	No
B1240	8551	10783	N/A	0.00237245, 0, 0.300842	0.00366619, 0.07977169, 0.290213	1.2543, 0.96119, 0.1486	Not significant	Elevation, potassium, frost period, mean temp, growing period, tmin1, tmax7	Sulfur, frost period, mean temp, growing period, tmin1	N/A	N/A	No
B1250	8600	59013	N/A	0.0104488, 0.1129688, 0.248594	0.00912798, 0.096248, 0.289	1.0664, 0.83597, 0.13265	1.2826, 0.84937, 0.18261	tmin1, prec6, prec7	Elevation, sulfur, frost period, mean temp, growing period, tmin1	N/A	N/A	No
B1415	9858	23064	N/A	0.00310502, 0, 0.331819	0.00531964, 0.08608937 ,0.315666	1.1673, 0.86677, 0.14448	Not significant	No significant associations	No significant associations	N/A	N/A	No
B1417	9880	25551	N/A	0.00397695, 0, 0.366737	0.00774632, 0.09401618, 0.26108	Not significant	Not significant	Weight/volume (soil), manganese, prec6, prec7, envPC1	No significant associations	N/A	N/A	No
B1423	9915	18176	N/A	0.00900549, 0.1129688, 0.271497	0.0133878, 0.1140064, 0.230989	Not significant	Not significant	No significant associations	Growing period, tmin1	N/A	N/A	No
B1450	10164	12478	N/A	0.0272767, 0.1843343, 0.203457	0.00641954, 0.0907386, 0.265038	Not significant	Not significant	No significant associations	Weight/volume (soil), pH (soil), exchangeable acidity (soil), potassium, sulfur, growing period, envPC3	N/A	N/A	No
B1568	11817	27587	N/A	Not significant	0.0406342, 0.1597317, 0.197582	Not significant	Not significant	envPC2	Weight/volume (soil), potassium, growing period, tmin1	N/A	N/A	No
B157	826	47908	N/A	0.0372081, 0.194094, 0.213639	Not significant	1.0967, 0.84797, 0.13601	Not significant	Elevation, potassium, mean temp, growing period, tmin1	Humic matter (soil), weight/volume (soil), potassium, sulfur, frost period, growing period, tmin1	N/A	N/A	No
B1574	11990	29209	N/A	0.00320761, 0, 0.313736	0.00119875, 0.07009153, 0.394852	Not significant	Not significant	No significant associations	Mean temp, growing period, tmin1	N/A	N/A	No
B1586	12502	72008	N/A	0.0101566, 0.1129688, 0.249305	0.0467566, 0.1644492, 0.182642	1.1517, 0.88478, 0.14046	Not significant	No significant associations	No significant associations	N/A	N/A	No
B1623	42670	76536	N/A	0.00117975, 0, 0.361835	Not significant	1.763, 0.9984, 0.22028	Not significant	Elevation, frost period, mean temp, growing period, tmin1, tmax7	No significant associations	N/A	N/A	No
B18	108	80060	N/A	0.000965384, 0, 0.376461	1.00E‐07, 3.32E‐05, 0.434547	Not significant	Not significant	No significant associations	Growing period, tmin1	N/A	N/A	No
B195	1034	7198	N/A	0.00114071, 0, 0.440777	0.0023812, 0.07107841, 0.289327	1.436, 0.9916, 0.17234	1.0625, 0.84957, 0.14688	Growing period, tmin1, prec7, envPC2	No significant associations	N/A	N/A	No
B233	1300	15568	N/A	0.00928923, 0.1129688, 0.252115	Not significant	1.2579, 0.96999, 0.14891	Not significant	Elevation, frost period, mean temp, growing period, tmin1, bio14, tmax7	No significant associations	N/A	N/A	No
B242	1392	7197	N/A	0.021182, 0.1527465, 0.220523	0.0459453, 0.1638531, 0.161433	Not significant	Not significant	Growing period, tmin1	envPC1	N/A	N/A	No
B244	1397	22957	N/A	0.000490435, 0, 0.402409	Not significant	1.1678, 0.89218, 0.14306	Not significant	Growing period, tmin1	No significant associations	N/A	N/A	No
B247	1439	41542	N/A	1.00E‐07, 0, 0.420581	0.00613931, 0.08849689, 0.333718	1.3083, 0.97399, 0.15595	Not significant	Frost period, growing period, tmin1, prec7, envPC2	Growing period, tmin1	N/A	N/A	No
B284	1697	40278	N/A	1.00E‐07, 0, 0.38978	0.00620887, 0.08849689, 0.288674	Not significant	Not significant	Weight/volume (soil)	No significant associations	N/A	N/A	No
B299	1824	75230	N/A	0.0424968, 0.194094, 0.185801	0.0388043, 0.1558586, 0.194044	Not significant	Not significant	Elevation, frost period, mean temp, growing period, tmin1, bio14, tmax7	Exchangeable acidity (soil), sulfur	N/A	N/A	No
B349	2240	78738	N/A	0.00720478, 0.1129688, 0.258533	Not significant	Not significant	1.2797, 0.92819, 0.17238	No significant associations	Manganese	N/A	N/A	No
B37	185	80930	N/A	Not significant	0.0357117, 0.1545958, 0.182394	Not significant	Not significant	Elevation, frost period	Elevation, tmax7	N/A	N/A	No
B372	2421	91530	N/A	Not significant	Not significant	1.2823, 0.95359, 0.15369	Not significant	envPC1	prec6, prec7	N/A	N/A	No
B447	3171	9594	N/A	0.0409416, 0.194094, 0.208842	0.0087962, 0.096248, 0.29196	Not significant	Not significant	Frost period, tmin1	Elevation, health score (1–5), weight/volume (soil), potassium, frost period, mean temp, growing period, tmin1, bio14	N/A	N/A	No
B573	3975	13709	N/A	1.00E‐07, 0, 0.431822	0.0127152, 0.1123806, 0.242335	1.2682, 0.9804, 0.14994	Not significant	Mean temp, growing period, tmin1	No significant associations	N/A	N/A	No
B594	4094	55798	N/A	0.0387994, 0.194094, 0.189429	0.00936509, 0.096248, 0.263922	Not significant	Not significant	No significant associations	Elevation, potassium, frost period, mean temp, growing period, tmin1, tmax7	N/A	N/A	No
B734	4908	40693	N/A	0.0237442, 0.1527465, 0.203394	Not significant	1.0929, 0.81996, 0.13773	Not significant	Elevation, frost period, growing period	No significant associations	N/A	N/A	No
B788	5255	36293	N/A	0.0189961, 0.1527465, 0.222877	Not significant	1.2484, 0.87097, 0.15556	Not significant	tmin1	No significant associations	N/A	N/A	No
B82	415	2453	N/A	Not significant	0.00520825, 0.08608937, 0.340989	Not significant	1.1612, 0.94659, 0.15342	No significant associations	Elevation, health score (1–5), weight/volume (soil), potassium, sulfur, frost period, mean temp, growing period, tmin1, bio14, tmax7	N/A	N/A	No
B841	5708	21745	N/A	0.0226805, 0.1527465, 0.227598	0.00146995, 0.07104953, 0.388837	Not significant	Not significant	Growing period, tmin1	Elevation, potassium, frost period, mean temp, growing period, bio14	N/A	N/A	No
B946	6496	23912	N/A	0.0138129, 0.1129688, 0.255514	Not significant	0.97626, 0.80596, 0.12303	Not significant	Weight/Volume (soil), envPC2	No significant associations	N/A	N/A	No
B977	6670	14487	N/A	1.00E‐07, 0, 0.589429	0.00155769, 0.07104953, 0.371962	1.6015, 1, 0.1911	Not significant	Weight/volume (soil), frost period, mean temp, growing period, tmin1, envPC2	Mean temp, tmin1, prec6	N/A	N/A	No
B999	6820	48285	N/A	0.018734, 0.1527465, 0.240148	0.0252617, 0.1394642, 0.226193	Not significant	Not significant	Growing period, tmin1	No significant associations	N/A	N/A	No

### 3.5 Detecting allele turnover patterns along ecological gradients: candidate vs. reference SNPs

**Figure 7:**
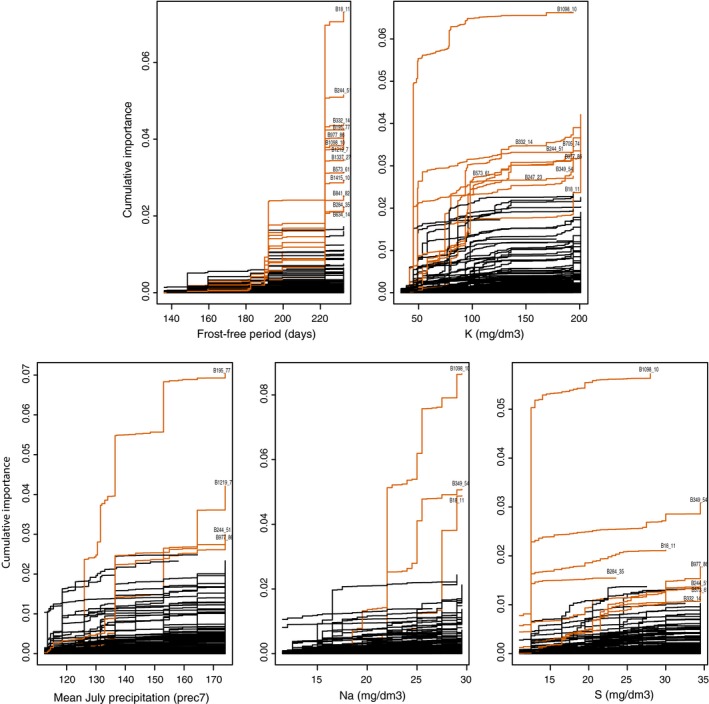
Gradient forest plots of SNP‐level compositional turnover. Highlighted and labeled functions indicate candidate SNPs with the highest cumulative importance, all of which retained signatures of being under selection when combined library of candidate and reference SNPs was reanalyzed with Arlequin, BayeScan, or LFMM. Functions labeled black are reference SNPs or candidate SNPs not contrasting from patterns of reference SNPs. Candidate and reference SNPs represented by 43 and 1,171 functions, respectively

The remaining set of environmental and functional trait variables with a VIF score below 10 were used for GF and mantel tests and listed in Table S3. After removing possible artifacts of combining libraries one and two (rationalized in Additional Validation of Environmental and SNP Data, Supporting Information), our chosen subset of 54 candidate loci was parsed to 43 in order to conduct GF on the combined dataset. The 43 candidate SNPs for GF analyses differed strongly from our chosen 1,171 reference SNPs (parsed originally from 1,307) in respect to overall patterns of allele turnover along ecological gradients of frost‐free period, mean July precipitation, and soil densities of potassium, sodium, manganese, phosphorus, and sulfur (Figure S9). In addition, mantel tests of collection sites for both reference and candidate SNP datasets supported that frost‐free period and levels of potassium were the top two ecological variables for explaining patterns of genetic differentiation, albeit isolation by distance was also strongly correlated (Table S3). For GF, individual allele functions of candidate SNPs were plotted and highlighted in Figure 7 against SNPs behaving as the genomic background (black line for background SNPs). The top five GF plots in Figure 7 showed the most overall contrast between individual candidate SNPs and reference SNPs (interpreted from Figure S9), but several patterns of allele turnover for less informative ecological gradients were of interest and were plotted in Figure S10. Several SNPs (e.g., B18_11, B244_51, B332_14, B195_77, B977_86) deviated greatly from reference SNPs near the longer portion of the frost‐free period gradient (highest allele turnover at approximately 220 days). SNP B1098_10 exhibited allele turnover patterns greatly contrasted from the majority of reference SNPs and other candidate SNPs along gradients of soil potassium, sodium, and sulfur. SNP B332_14 ranked second for a high amount of turnover along a potassium gradient, and SNPs B349_54 and B18_11 were also strong candidates that exhibited contrasting allele turnover patterns along gradients of sodium and sulfur. For allele turnover patterns along precipitation gradients, SNPs B195_77 and B1219 were highly contrasted against patterns of the reference background and other candidate SNPs.

## 4 Discussion

This study characterized how *C. florida* might have evolved local adaptation in response to a heterogeneous landscape of ecological pressures within mountain, Piedmont, and coastal regions of North Carolina. While further study along the entire range of *C. florida* is ongoing, our conclusions of the adaptive variation in North Carolina may be related to findings of the broader species range. North Carolina has long been noted to contain a large variety of plant communities (i.e., Southern Appalachian forests, savannah, pocosin, and swamps), which are similar in species composition to communities at more northern and southern latitudes (Wells, 1932). There is still a sizeable portion of adaptive variation uncharacterized in this study when compared to the adaptive variation present in the broader range (Table S4). Nonetheless, our results are highly relevant to conserving the species in Southern Appalachia, and our assessment of repeatability can aid efforts to identify consistent and reliable candidate loci as additional sequence libraries are incorporated.

The relationship between plant community composition, species occurrence, and ecological gradients have been extensively examined in the Carolinas (Peet et al., 2012), but the genetic basis for intraspecific turnover along ecological gradients has been less understood. Environmental processes leading to local adaptation have also been frequently overlooked in studies pursuing candidate SNPs of local adaptation (Meirmans, 2015). Our association study of *C. florida* populations from three divergent environments provided such insights, in addition to uncovering where population‐level vulnerability to climate change and exotic disease (Anderson et al., 2004; Liebhold, Brockerhoff, Garrett, Parke, & Britton, 2012; Pautasso, Döring, Garbelotto, Pellis, & Jeger, 2012; Weed, Ayres, & Hicke, 2013) might occur. Our results’ specific implications for conservation of the flowering dogwood tree are shared in Supporting Information (Implications for Conservation), while we focus on particular candidate loci and ecological pressures in our discussion. We also report the repeatability of our GBS experiments in respect to the small subset of candidate SNPs that are consistently associated with local adaptation across both libraries and are cross‐examined across multiple selection models (Villemereuil et al., 2014).

### 4.1 Evidence for locally adapted candidate loci

Local adaptation could be inferred from genetic signatures intrinsic to sequence datasets or allele frequency changes in relation to functional and environmental traits; Schoville et al. (2012) reviewed excellent examples of both population genetic and GEA approaches to uncover local adaption. Using both approaches, we found evident genetic signatures of local adaptation in *C. florida* (Table 3) and 54 well‐supported candidate loci under selection along divergent environmental gradients (Figure 7). The majority of the candidate loci (42 of 54; Figure 6b) still showed evidence of being under selection when the two sequence libraries were combined, validated further (Additional Validation of Environmental and SNP Data, Supporting Information), and reanalyzed with outlier tests and association models. Support for biological significance of several SNPs was clear when various criteria of candidate status were examined together. Moreover, select ecological pressures appeared to influence candidate loci. Three candidate loci (B332, B1350, B982) were selected for further consideration from a set of putative loci under selection (Table 3) based on compelling predicted functions. SNPs that did not necessarily have annotated function but showed strong splits along ecological gradients where allele turnover was high (according to GF analysis) were also noted (Evidence for Locally Adapted Candidate Loci, Supporting Information).

Patterns of overall cumulative importance—or how well biological variation was explained for a given interval of environmental change (Fitzpatrick & Keller, 2015)—from GF analyses (Figure S9) showed candidate SNPs were most divergent from reference SNPs for gradients of frost‐free period, July precipitation, potassium, phosphorous, sulfur, and sodium. GEA results from LFMM also supported the importance of these variables for explaining local adaptation in specific loci. As summarized in Figure 5, temperature covariates (tmin1, growth period, mean temperature, and frost period) constituted the majority of association results to individual SNPs. Soil characteristics with highest amounts of significant LFMM associations were potassium levels and envPC2, which was a reduced‐representation component mainly characterizing soil differences from an environmental distance‐based PCA (Figure S3). Low potassium levels were characteristic of relatively acidic soils of the Coastal Plains, which were susceptible to soil leaching and deficiencies in other plant nutrients (USDA soil classification). In conjunction with soil nutrient availability, the amount of moisture available to soils clearly impacted nutrient use and growth strategies in flowering dogwoods (Kost & Boerner, 1985), and temperature and moisture regimen were shown to affect disease prevalence in dogwood populations (Chellemi et al., 1992). Holzmueller et al. (2007) demonstrated deficiency in soil potassium was linked to higher severity of dogwood anthracnose and quicker infection rates. The findings of Holzmueller et al. (2007) were of particular interest as coastal trees we sampled grew in relatively potassium poor soils but appeared visually free of anthracnose disease. This suggested coastal trees might have adapted to potassium poor soils, which could have resulted in secondary fitness effects such as greater resistance to disease relative to montane populations. In other words, selective pressures regarding levels of soil potassium might play a role in the adaptation to osmotic stress in coastal populations, and any such adaptations might predispose coastal populations to be less susceptible to oxidative stress caused by disease. Dogwood anthracnose was reported in Dare County along North Carolina's coast (Figure S1), but its failure to persist there also suggests the pathogen might not be adapted to thrive in North Carolina's coastal climate. As such, adaptive mechanisms linking drought tolerance to disease resistance remain highly speculative—requiring us to test such hypotheses in future experiments.

The SNP on locus B332 (B332_14) was one of only nine genomic locations (B977, B768, B757, B634, B332, B247, B195, B1240, and B1250) to meet five of the six criteria for being considered a candidate of selection. It is repeatedly identified as a high *F*_st_ outlier by Arlequin in both the first, second, and combined libraries and by BayeScan in library two and the combined library. LFMM analyses in both libraries and the combined library revealed the SNP was significantly correlated to several climate and soil variables, namely potassium and a reduced‐dimension environmental variable (envPC2) autocorrelated with soil measures. We speculated soil leaching pressures and plant adaptations for efficient regulation of osmoticum might be critical factors to explain the high turnover of alleles for this SNP along a potassium gradient (Figure 7). B332_14 aligned to an exon of *C. florida*'s transcriptome (Table S1), and the transcript was predicted to encode a probable glycerol‐3‐phosphate dehydrogenase (GPD, accession: XM_006493426) (Table 3). As demonstrated by Albertyn, Hohmann, Thevelein, and Prior (1994) and later by Shen, Hohmann, Jensen, and Bohnert (1999), the efficiency of proteins to regulate levels of glycerol influenced the ability of plants to osmotically adjust to salt stress and other osmotic stresses tied to water flux. Coastal trees we sampled grew in soils that had significantly lower levels of potassium (Table S1 Appendix), and they most likely had acquired locally adapted mechanisms to compensate for lower potassium uptake and resulting toxicities of high proportions of sodium to potassium (Niu, Bressan, Hasegawa, & Pardo, 1995). The GF plot of potassium (Figure 7) supported strong turnover of alleles for B332_14 along lower levels of potassium. Supplementary GF analyses (Figure S10) supported gradual turnover of the biallelic locus as plant osmoticum (measured in osmometer experiments) increased (Functional Traits, Supporting Information). While one allele was more predominant in populations of the Piedmont and Coastal Plains of North Carolina, the alternate version was only the major allele in two of the four mountain sites where soils were relatively potassium rich (Table S1). Of the 1,171 reference and 43 candidate SNPs analyzed by GF, only 24 SNPs had a correlation threshold above 0.5 in comparison with osmometer readings, but SNP B332_14 had the second highest cumulative importance of those 24 SNPs. SNP B332_14 also had the second highest cumulative importance along a gradient of overhead canopy cover, further suggesting it might be involved in managing other sources of osmotic stress such as high light intensity. Thus, this is a strong candidate associated with local adaptation of the species.

Locus B1350 is predicted to be a leucine‐rich repeat (lrr) receptor‐like serine threonine–protein kinase (accession: XM_00648094). While the function of this particular locus is speculative, leucine‐rich repeat receptor‐like kinases have previously been implicated in pathogen recognition (Afzal, Wood, & Lightfoot, 2008). A variety of resistance (R) genes encoding for lrrs have been identified, as the domain normally interacts with pathogen effectors or intermediate host molecules to trigger plant responses (Caplan, Padmanabhan, & Dinesh‐Kumar, 2008). One allele of B1350 was highly abundant in two of the four mountain subpopulations where dogwood disease was greatest (Table S1). Within analyses of library one, this locus was detected as a candidate of selection by both Arlequin, BayeScan, and LFMM. Greater heterogeneities of elevation, slope, and temperature within the mountains might result in more independent and isolated cases of local adaptation than would be expected within the same spatial scale for nonmountainous regions. Moreover, as implied by Hadziabdic et al. (2012), some mountain populations of *C. florida* might escape the suitable habitat range of dogwood anthracnose and maintain high genetic diversity instead of having advantageous alleles become fixed. Our identification of a lrr repeat receptor kinase constituted one hypothesis of local adaptation to disease for two of our sampled subpopulations, but we also found another putative R gene in our candidate dataset that might represent a different mechanism of plant resistance (discussed below).

Locus B982 was designated a candidate locus in analyses of both sequence libraries as well as the combined library. While the locus’ change in alleles was not associated with any ecological gradients, it was highly differentiated in both libraries and had been detected as an outlier by both Arlequin and BayeScan analyses in the combined library. Moreover, locus B982 aligned to a sequence scaffold of a coding region within the transcriptome of *C. florida* (scaffold 19651, Zhang et al., 2013). The scaffold itself aligned to a gene predicted to encode a lectin protein kinase. While extracellular signaling of lectin protein kinases might be specialized for various extracellular signals, an emerging role of such proteins might involve innate immunity responses in plants (Singh & Zimmerli, 2013). One allelic version of this biallelic locus was fixed or nearly fixed in most of the populations sampled. However, consistent estimation of allele frequency in both libraries suggests the minor allele existed in the Umstead population (Piedmont) at an approximate ratio of one to ten and in the Croatan (a coastal population) at a one to three ratio. We suggested that any local adaptation associated with the minor allele of B982 might be the result of a relatively recent mutation originating in a small area of transition from Piedmont to Coast. Future examination would be necessary to determine whether locus B982 was responsible for local adaptation, a deleterious susceptibility phenotype, or was related to demographic histories of more southern populations along the Atlantic Coastal Plains. Moreover, as our disease incidence scores are confounded with the adaptive landscape of other abiotic pressures and predictions of our annotated R genes have not been confirmed by functional experiments, we reiterate that the few loci associated with plant health must be interpreted with caution.

### 4.2 Repeatability of detecting putative loci under selection

Repeatability has long been a concern for all genotyping methods, including next‐generation sequencing methods (Crawford, Koscinski, & Keyghobadi, 2012). We focused specifically on detection of candidates under selection using double‐digest GBS. The sequencing method has remained valuable for detecting genetic signatures of selection on individual genotypes because of its low cost and high yield of genetic markers. However, researchers were recently recommended to incorporate sequencing replication in their experimental design for initial pilot experiments of GBS (Mastretta‐Yanes et al., 2015). Restriction site polymorphism and stochastic sequencing processes related to fragment length might lead to preferential genotyping in certain loci or individuals and result in missing data that biases downstream analyses of GBS libraries (Gautier et al., 2013). Without proper filtering of loci and minor alleles along with additional validation of SNPs, missing data might increase false‐positive rates for identifying highly differentiated outlier loci. Even with filtering, concern remains that if populations were sequenced and analyzed again, many previously identified candidate loci would not be detected in subsequent analysis due to stochastic variation in sequencing, stringent filtering criteria that removes loci in libraries with lower average sequencing depth (Mastretta‐Yanes et al., 2015), and the possibility that newly sequenced samples lack some restriction sites in samples from others datasets (Arnold, Corbett‐Detig, Hartl, & Bomblies, 2013). These factors might substantially bias results of population genetic analyses with GBS data depending on filtering criteria, especially in regard to detection of SNP outliers.

To address the aforementioned concerns, we repeated GBS experimentation with an independent library of Illumina sequencing using different randomly selected individuals of the same populations. Our goal was to see whether congruent results from various analyses would be obtained using data from DNA libraries of two experiments. We obtained similar population demographic results. For instance, average observed heterozygosity of populations was consistently estimated to be 0.26, and average nucleotide diversity among the populations was estimated to be approximately 0.29 in both libraries. STRUCTURE and AMOVA tests consistently showed about 3% of genetic variation could be attributed to coastal vs. mainland group definitions, while the majority of genetic variation was attributable to differences between individuals regardless of population, providing additional evidence for the genetic consequences of bird dispersal of flowering dogwood fruits (Call et al., 2015; Hadziabdic et al., 2010). While demographic trends were relatively consistent when compared across both GBS datasets, there was only a small portion of candidate loci consistently detected between analyses of the two sequence libraries (Figure 6a), albeit the 54 loci considered candidates of selection (Table 3) showed largely consistent patterns of changes in allele frequencies among populations sampled (Table S1).

The total genotyped loci shared among library one and two ranged from approximately 75%–82% when compared to the opposing library, but the percentage of loci consistently identified to be under selection dropped to a range of approximately 23%–32% (see Defining Consistency, Supporting Information). Of the 43 candidate loci consistently showing evidence of selection among the two libraries after additional SNP validation (Additional Validation of Environmental and SNP Data, Supporting Information), all but one of the candidates (locus 5B) still showed evidence of selection when the combined library was reanalyzed (Figure 6B). The differences between the rate of consistently genotyping a locus and the rate of consistently identifying the locus to be under selection could be a result of sequencing inconsistency, differences in library quality, and differences in genotypes of individuals included in the two experiments (although from the same populations). Furthermore, both of the libraries were run with samples of other sequence experiments in different lanes, resulting in some mean differences of total utilized reads per sample. For instance, the average number of sequence reads per sample in library one was approximately 860,000 while the average was approximately 1.17 million for library two. Additionally, sequencing efficiency differences across the two different flow cells might have resulted in slight mean differences in quality score. Some heterogeneity among samples could have occurred at the PCR enrichment step, and there might also be uncharacterized variation in genome size among natural populations, which could result in variation of total sequence reads among samples. Nonetheless, mean depth coverage per individual remained on average above 30×, which according to precedents aiming for an average of 20× coverage per RAD‐tag (Malinsky et al., 2015), might be sufficient to avoid most instances of allele dropout. Moreover, experimental and analytical procedures implemented in this study were designed to minimize downstream effects of the potential biases described.

In regard to library quality, we minimized biases using high‐quality DNA of the same amount for each sample, and we selected fragments of 300 ± 36 bps for sequencing. Although sampling size might influence results, the sample size of our subdivided populations was still sufficiently large to accurately make population genetic inferences, according to simulated study (Buerkle & Gompert, 2013). Despite potential biases and their causes, our approach placed greater credence in results that were consistent between the two libraries. This approach resulted in us considering a smaller number of locally adapted candidate SNPs, but it reduced false positives as demonstrated in Figure 4. A candidate SNP detected by analyses of data in one of the two libraries might either be a false positive due to highly differentiated sequence error or a true candidate locus for selection. Results from our comparison of two GBS libraries suggested caution in interpreting the adaptive significance of loci that were found to be highly differentiated in only one library. In order to flag loci as candidates under selection with higher certainty, we recommended experimental repetition and the use of multiple analyses, including various *F*_st_ outlier tests, GEA tests, and novel methods like GF.

## 5 Conclusions and Future Directions

Our GBS study of six populations of *Cornus florida* from three divergent ecosystems representing the Atlantic Coastal Plains, Piedmont, and southern Appalachian Mountains found evidence of local adaptation. Several soil nutrients (K, Na, and P) and temperature during the growing season were important drivers of ecological and genetic divergence of the species. The study identified 54 putative candidate loci under selection for local adaptation. A few had annotated functions in biological processes that might have adaptive roles such as increased hardiness to drought and disease resistance. Several of these loci will serve as candidates for a broader scale and more thorough analysis to further support roles for genes of interest. We concluded high genetic variation within populations and significant allelic differences among ecologically heterogeneous regions readily predisposed *C. florida* for local adaptation to ongoing climatic shifts. We expect some independent local adaptations to occur in other areas outside this pilot study's scope, but the genetic and ecological patterns detected here have provided hypotheses for an expanded study that will assess large‐scale environmental gradients and genetic structure across the species range.

## Conflict of Interest

None declared.

## Data Accessibility

Environmental and physiological data available in Dryad Digital Repository: doi:10.5061/dryad.h55q8. GBS datasets for each sequence library along with adaptor–barcode information are available as separate files in Supporting Information (Data S1–S3) in addition to metadata corresponding to analyses of each locus (Table S1).
