# Supplementary material for: Ecological genomics of local adaptation in Cornus florida L. by genotyping by sequencing
Source: Ecol Evol. 2016 Dec 20;7(1):441–65. doi: 10.1002/ece3.2623 (PMC5213257; doi:10.1002/ece3.2623)
Supplement: Supplementary file 2 [file ECE3-7-441-s002.docx]

**Supporting Information**

*Flowering Dogwood Background*

Mountain populations of *C. florida* in the southeastern United States have declined 48-98% in various sites since the introduction of dogwood anthracnose disease (Sherald *et al* 1996; Hiers and Evans 1997; Williams and Moriarity 1999; McEwan *et al* 2000; Rossell *et al* 2001; Jenkins and White 2002). If *C. florida* populations were to continue declining, decreasing amounts of calcium-rich leaf litter from *C. florida* (Baird 1980; Blair 1982; Linzey and Brecht 2003; Borer *et al* 2013; Lovenshimer and Frick-Ruppert 2013) could lead to lower buffering capacity of the forests in already high-acidic soils of the southeast, exacerbate invertebrates’ ability to thrive in soils, and change forests to become unfavorable for frugivorous birds (Rhoades *et al* 2011a; Rhoades *et al* 2011b). Therefore, saving the species has profound significance on the healthy function of forest ecosystem in the mountains. In addition to ecological importance, the species has traditionally been valued in horticulture and ornamental markets due to its spectacular inflorescences in the spring and red leaves and fruits in the fall—netting up to 30 million dollars in annual sales (NASS, USDA. Census of Agriculture 2007).

*Defining Consistency*

To clarify our definition of consistency, take for example the consistent genotyping across libraries. When we set our filtering criteria to retain loci with less than 25% missing data across samples in a given library, we found that 75% of the loci from library one met the same criteria as in library two. Similarly, of loci from library two that met this criteria (having loci with less than 25% missing data as in library two), 82% of loci met the same criteria as in library one. To clarify our definition of a locus being a consistently detected candidate under selection, take for example the number of loci in library one that were consistently detected to be candidates of selection. Twenty-three percent of the loci identified to be candidates of selection in library one also met consistent genotyping criteria in library two (only considering loci passing filtering constraints) and were also detected to be candidates under selection in library two.

*Environmental Variables (continued)*

Elevation of a sampled tree was determined by GPS. Proximity to water (whether a sampled tree was within visual distance of a pond, stream, river, or lake) was recorded as binary data. Canopy coverage was estimated as the average percentage of canopy from opposing cardinal directions covering a given tree (measured by densiometer). Soil surface cores were taken around every tree sampled. The soil samples from nearby trees of similar microhabitat were pooled together. Pooled samples were left dry at room temperature and delivered to the North Carolina Department of Agriculture & Consumer Services’ Agronomic Division for measurement of phosphorous, potassium, calcium, magnesium, sulfur, sodium, manganese, copper, zinc (mg/dm3), and percent humic matter (HM), weight to volume ratio of soil, cation exchange capacity (CEC), portion of CEC composed by hydrogen and aluminum (exchangeable acidity or Ac), soil pH, and percent base saturation. For each subpopulation, the soil characteristics were represented by multiple sets of pooled soil cores.

*GIS Resources*

We used the following geospatial information system (GIS) resources: Soil Survey Geographic Database (SSURGO), Parameter-elevation Regression on Independent Slopes Model (PRISM), U.S. Geological Survey (USGS) bioclimatic predictors (O’Donnell and Ignizio 2012), International Institute for Applied Systems Analysis and Food and Agriculture Organization’s (IIASA/FAO) length of growing period data, and Average Growing Season for selected North Carolina locations (1951-1980) reported by North Carolina’s Cooperative Extension Service (1998). Climate data were obtained from records from 1950-2000 ([www.worldclim.org](http://www.worldclim.org); Hijmans *et al* 2005) using ArcGIS. The following variables were extracted for each GPS location of sampled individuals: mean precipitation in June (prec6) and July (prec7), precipitation of driest month (bio14), mean monthly precipitation year-round (bio12), minimum temperature of January (tmin1), maximum temperature of July (tmax7), and mean annual temperature (bio1). Frost free period and length of growing period predictions were modeled based on several bioclimatic predictors including temperature and precipitation, but records of average growing season were directly obtained from weather stations nearby sampling sites.

*Functional Traits (Osmometer continued)*

The partially submerged cuttings were allowed to undergo osmotic adjustment in solution by storing them in dark conditions overnight. Cuttings were allowed 24 hours to reach equilibrium with water. By allowing each cutting to reach equilibrium with the water solution under standardized conditions, we were able to measure and compare leaf osmotic potential of individuals when leaves were at full turgor. Eight leaves in full turgor were randomly selected and hole-punched from each cutting and measured for solute molarities or osmolality (inversely related to osmotic potential) using an osmometer. Measurements were taken repeatedly (approximately an average of six times) until readings were stable. In other words, repeated measurements were taken until the difference between subsequent measures was below or equal to ten mmols/kg to ensure that the osmometer chamber reached equilibrium with the ambient temperature during its analysis of each sample. Since previous studies have established the inverse relationship between leaf osmotic potential and leaf osmolarity (Gebre *et al* 1998; Tschaplinski *et al* 1998), we treated the osmolality measurements as representative data of leaf osmotic potential. This association permitted us to posit trees with high osmolality (high cytosol concentration and large negative leaf osmotic potential) can more efficiently retain water content in cells compared to trees with low osmolality.

*Genotyping (continued)*

Ninety-six unique oligonucleotide barcodes were designed for paired end one (PE1) adaptors and were used in conjunction with five uniquely designed paired end two (PE2) barcode adaptors (Data S3) to allow multiplexing of 96 and 85 individuals in the pooled libraries and provide comparable nucleotide composition in the barcodes at the beginning of Illumina Hiseq runs. 750 ng of DNA from each sample in the set was digested by PstI and MspI endonucleases. Barcode adaptors were then ligated to restriction enzyme-digested DNA fragments with unique PE1-PE2 barcode combinations for each sample. Adaptor-ligated products from each DNA sample were pooled and purified using a Qiagen PCR purification kit. Eight PCR reactions were performed using the pooled DNA as templates and adaptor-specific primers. The eight PCR products were pooled together and size selected using Pippen Prep technology for 300 + 36bp fragments before submission for 100 base pair paired-end sequencing by service of NCSU’s Genomic Science Lab.

*Paired-end two (PE2) reads*

The 80bp PE2 reads were shorter than the 90bp PE1 reads and sometimes overlapped with PE1 reads. Since a reference genome of *Cornus florida* was still in development, we were not able to utilize alignment reports to determine if a PE2 read overlapped with the same locus of a PE1 read. Therefore, reported results on population genetics and candidate loci were based on analyses of PE1 data.

*SNP Data Processing (continued)*

We justified three identical raw reads as a minimum to create a stack (default) because setting the threshold too high could artificially result in allele dropout if heterozygous loci are incorrectly called homozygous (Catchen *et al* 2013; Mastretta-Yanes *et al* 2015). The rationale for choosing the remaining parameters for the STACKS platform was three-fold. First, repeated empirical tests of option combinations were done using as many stringent filtering settings as possible before the number of SNPs in our dataset decreased substantially. Second, recent findings on the optimal STACKS parameter ranges for a plant species with similar genome size and PE read length (Mastretta-Yanes *et al* 2015) showed that our settings did not deviate substantially from their optimal option settings. Third, our mean depth of coverage for both libraries was slightly above 30x (Table 1), larger than the coverage used by other studies such as Malinsky et al 2015. Given these precedents, we determined that our options were sufficient if the main criterion was to obtain a conservative yet large enough dataset for population genetic analyses of SNP markers.

*Additional Validation of Environmental and SNP Data*

We implemented one more series of validation steps to our environmental and SNP datasets prior to GF analysis, mantel tests, STRUCTURE analysis, and PCA of combined library. GF analysis was sensitive to collinearity among environmental variables, and combining genetic datasets from two libraries without additional filtering could produce artifacts as a result of differences in sample size and coverage between libraries. As a standard procedure to reduce collinearity within our environmental dataset (Hair *et al* 1995), we used a stepwise procedure (<http://www.r-bloggers.com/collinearity-and-stepwise-vif-selection/>) to calculate and remove variables until all remaining variables had a VIF score below 10. For our genetic datasets, we re-implemented our minor allele frequency filter cutoff of 5% for the combined sample matrix and passed a slightly more stringent missing genotype acceptance threshold (20% instead of 25%) after combining library one and two datasets for the group of candidate and reference loci. As an additional safeguard to reduce artifacts from combining sequence libraries with different mean coverages and possible artificial excesses of homozygotes for the library with lower coverage (Mastretta-Yanes *et al* 2015), we removed loci out of Hardy-Weinberg equilibrium in more than four subpopulations for our combined dataset according to exact tests using Genepop version 4.2 (Rousset 2008). After implementing these validation steps on the genetic datasets for GF and mantel tests, the number of available RAD-tags was reduced from 54 to 43 for the candidate group and 1,307 to 1,171 for the reference group. Additional reduction of SNP data was done during the fitting procedure of GF models, depending on how many SNPs met the correlation threshold of 0.5 for a given gradient.

*Evidence for Locally Adapted Candidate Loci (continued)*

Most candidate loci identified did not have clear predicted functions linking to mechanisms for local adaptation but exhibited signs of natural selection such as exceptional genetic differentiation beyond the expectations of neutral genetic structure or significant associations to functional-ecological variables. Several loci (B1092, B1219, B768, B757, B124) aligned to housekeeping genes that were orthologous in other plants, but only B1219 (nucleotide binding GO, accession: XM_010110700) displayed substantial allele turnover along a gradient of mean temperature in July. Many candidate SNPs with unknown functions showed strong associations to environment and were highly differentiated among populations. For instance, the SNP located on locus B1098 was consistently called as an *Fst* outlier and exhibited the highest amount of allele turnover along a 22.5 to 30.0 mg/dm3 gradient of sodium when compared to all other reference and candidate SNPs. Some of these SNPs like B1098 were also identified as associated with other ecological gradients (i.e. potassium and sulfur) according to LFMM and GF analyses, suggesting ecological pressures related to various soil nutrients and climatic variables might act in complementary ways to drive allelic turnover for a given SNP. Results from GF analysis, however, revealed that sodium levels might be more important and best explain allele turnover of for the B1098 locus; the GF function for sodium and B1098 had the greatest cumulative importance value compared to the GF plots of potassium and sulfur, and the allele turnover pattern along a sodium gradient spanned multiple geographic locations.

*Implications for Conservation*

Our findings of high allelic turnover along ecological gradients provide guidance on prioritizing areas for conservation. While conservation efforts should preserve and protect suitable habitats for the species, resources could be dedicated to habitats that exhibit the range of variation associated with the species’ genetic variation. Monitoring intraspecific genetic variation using GBS data at larger landscape-scales across the species range would be useful for developing a comprehensive strategy for species conservation. For instance, our results provided valuable insights in support for identifying and reserving potassium-rich habitats in the mountains for purposes of populating and preserving the species native to that region. Furthermore, our results indicated that coastal region genotypes (better adapted to poor potassium soil than mountain populations) could be transplanted in the mountains without substantially reducing frequencies of mountain genotypes since the species appears to be genetically well-mixed by high levels of gene flow.

Although genetic differentiation between the coastal and mountain-Piedmont regions was detected, the level of genetic variance attributed to differences between the two groups was relatively low—only three percent (Table 2). While the southeastern Atlantic Coastal Plains region was not sampled in Call *et al* 2015 (the most recent study of natural populations of *C. florida* using chloroplast genes), the level of differentiation between ecoregions defined in that study (Hot Continental, Hot Continental-Mountain, and Subtropical) were comparable (4.29%). We found little evidence of SNPs correlated to disease symptoms, but we noted loci that were potentially involved in disease responses. B1350 was one such candidate locus, suggesting that the species was under selection at or near this locus and that the surrounding genomic region should be monitored in other populations to designate conservation status of individuals with the genotype of interest. Furthermore, there might be more private alleles unique to the populations of the southeastern Atlantic Coastal Plains because they occur on the eastern edge of the species’ range (Maggs *et al* 2008). Therefore, such unique alleles may have higher conservation value if the goal is to enrich declining flowering dogwood populations with alleles novel to a given region—ensuring dogwood’s functional role in forest ecosystems persists. Lastly, the candidate SNPs we discovered could be valuable for selecting germplasm for cultivar improvement programs that wish to breed traits related to disease resistance and hardiness in particular climates. Candidate loci uncovered from our pilot study will be aligned to a draft genome in progress so we can further target proximate genomic regions of interest and genotype across populations of high conservation value.

***Supplementary Tables and Figures***

**Figure S1.** Subset of collections (bottom) from broader study of *C. florida* (top) applied to this study of North Carolinian populations. Red counties have known occurrence of dogwood anthracnose disease. For subset of populations sampled in this study, differences in mean monthly rainfall, length of growing period, soil type, and county occurrence of dogwood anthracnose are visualized to demonstrate the heterogeneity in environment that exists among the mountain, Piedmont, and Coastal Plain ecoregions of North Carolina.


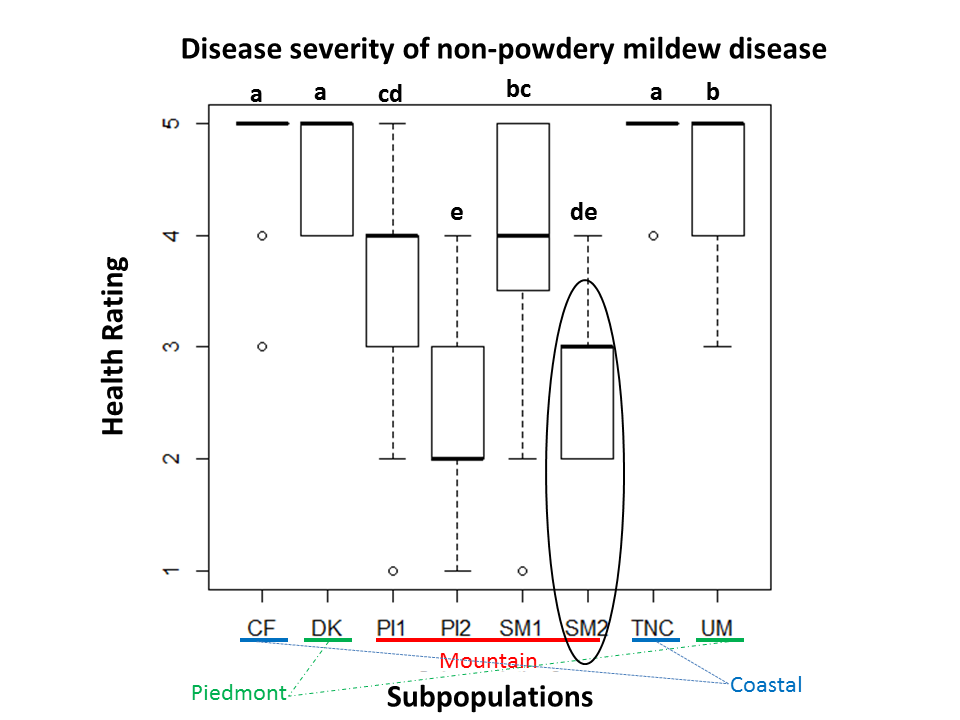


**Figure S2.** Box and whisker plots showing minimum, maximum, quantiles, and median distributions of health scores across subpopulations. Ecological regions and subpopulation abbreviations listed at bottom of plot, and results of Dunnett modified Tukey-Kramer results listed at top (same letters indicating non-significance of compared groups). Circled subpopulations represent collection sites confirmed by North Carolina’s Forest Health Monitoring services to have dogwood anthracnose.


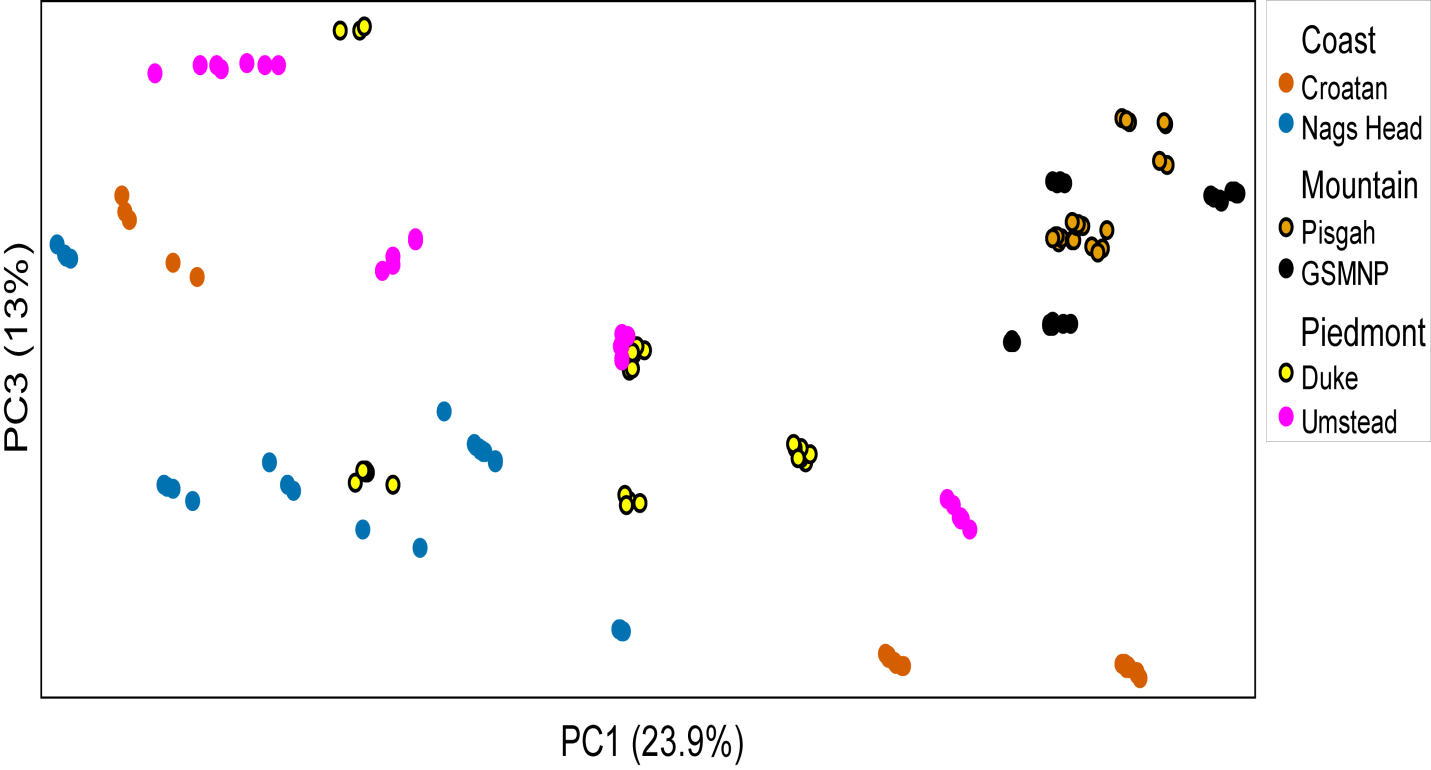


**Figure S3.** PCA of samples derived from 28 standardized environmental variables. After removing missing values from matrix, PCA scores of 174 samples from both libraries were calculated and plotted along the two principal components (PC) explaining the most variance.

**
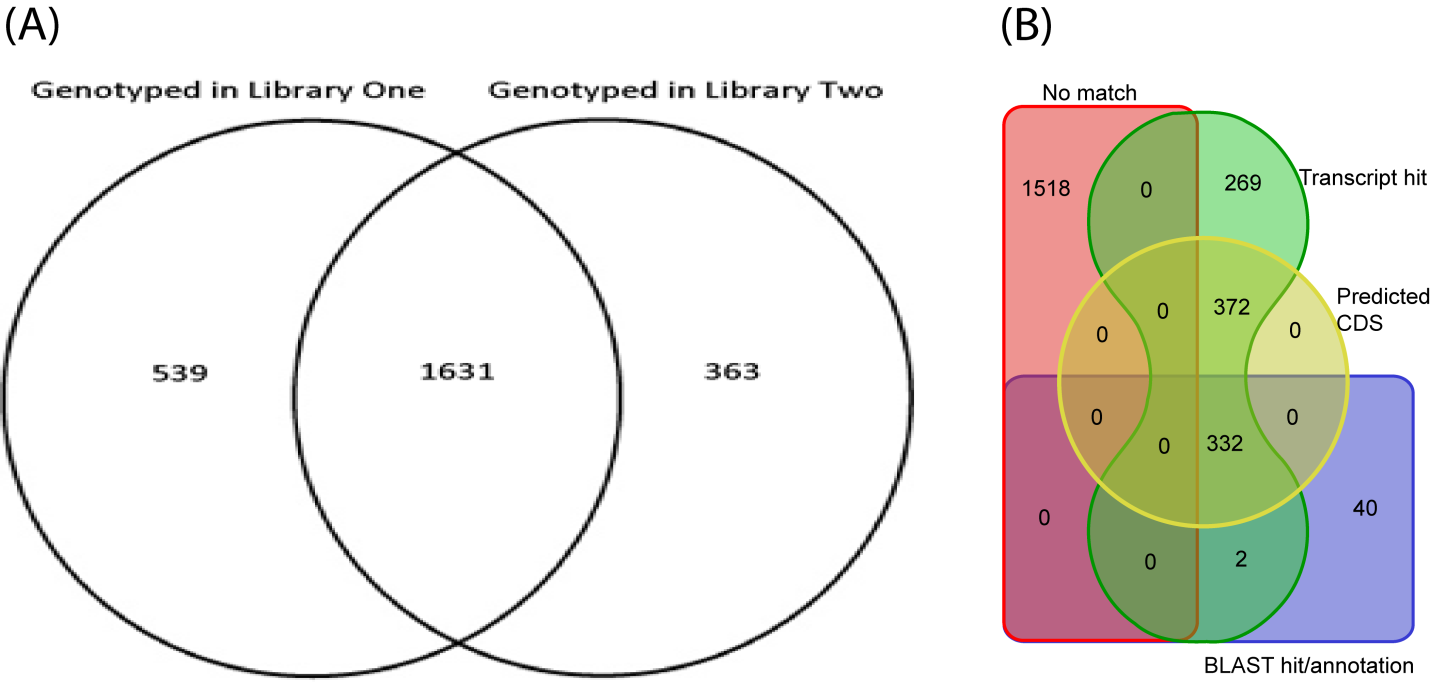
**

**Figure S4.** Venn diagram representing (A) RAD-tag loci genotyped consistently among libraries one and two and RAD-tags unique to library one and two as well as (B) total unique loci with BLAST hit-annotations or matches to mRNA transcripts or coding regions (CDS) of *C. florida.*


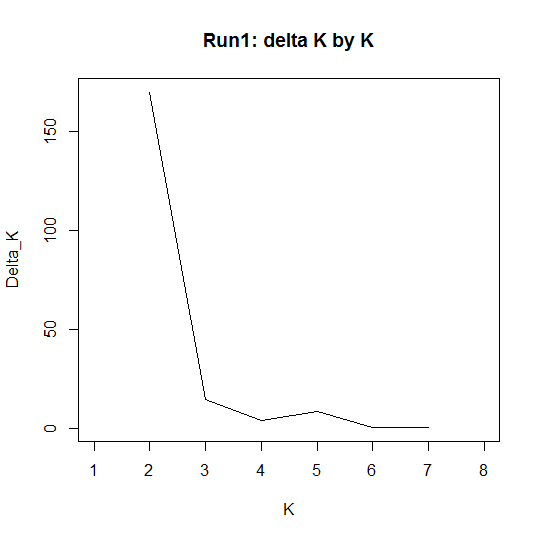


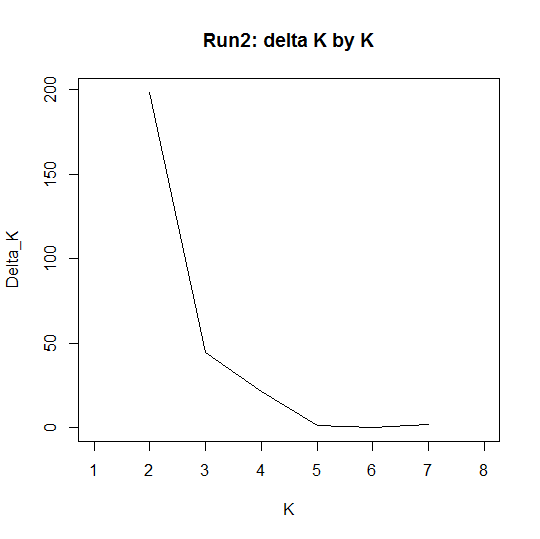


**Figure S5.** Evanno plots of STRUCTURE analyses for library one and two. First eight cluster models depicted from STRUCTURE analyses with ten replicates per K value, a burn-in of 100,000, and 100,000 subsequent iterations. Sequence datasets parsed of correlated loci pairs via removal of multiple SNPs per RAD-tag.


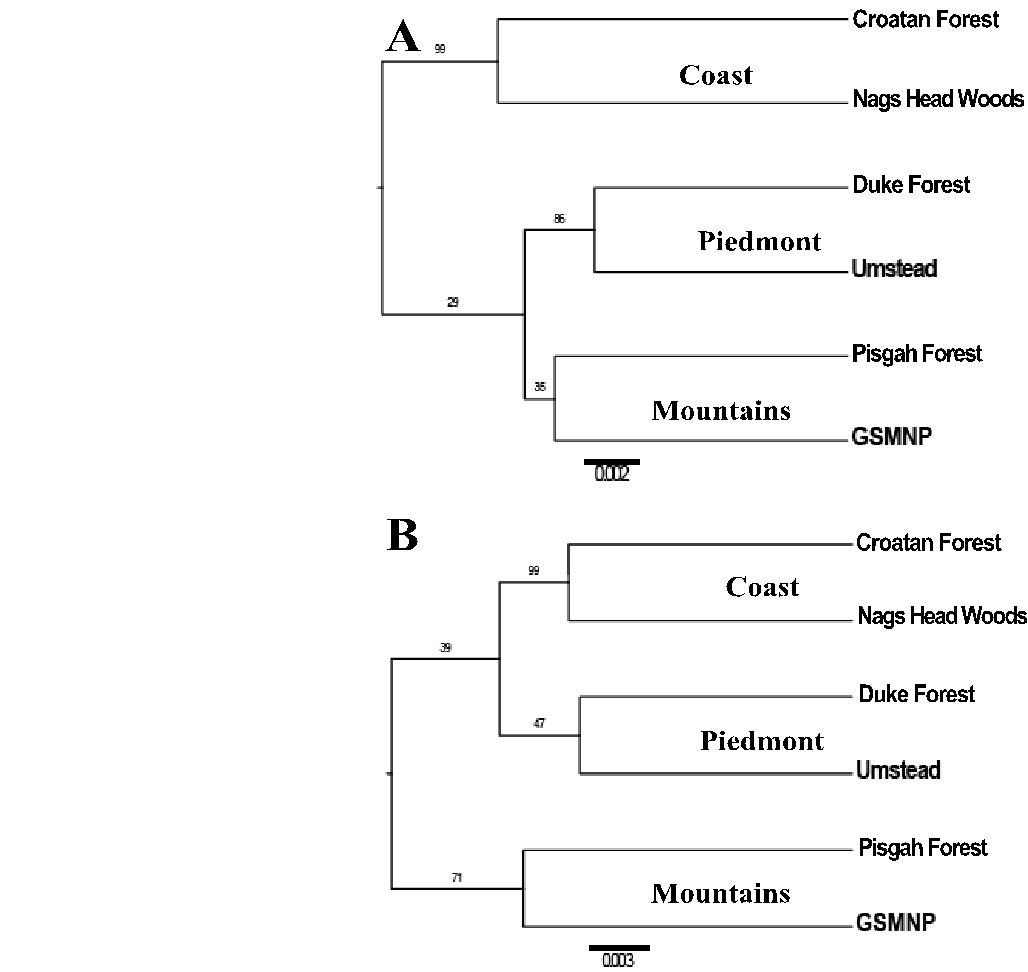


**Figure S6.** UPGMA results of (A) library one and (B) library two using Nei’s standard genetic distance (Nei 1972) and 100 bootstraps.


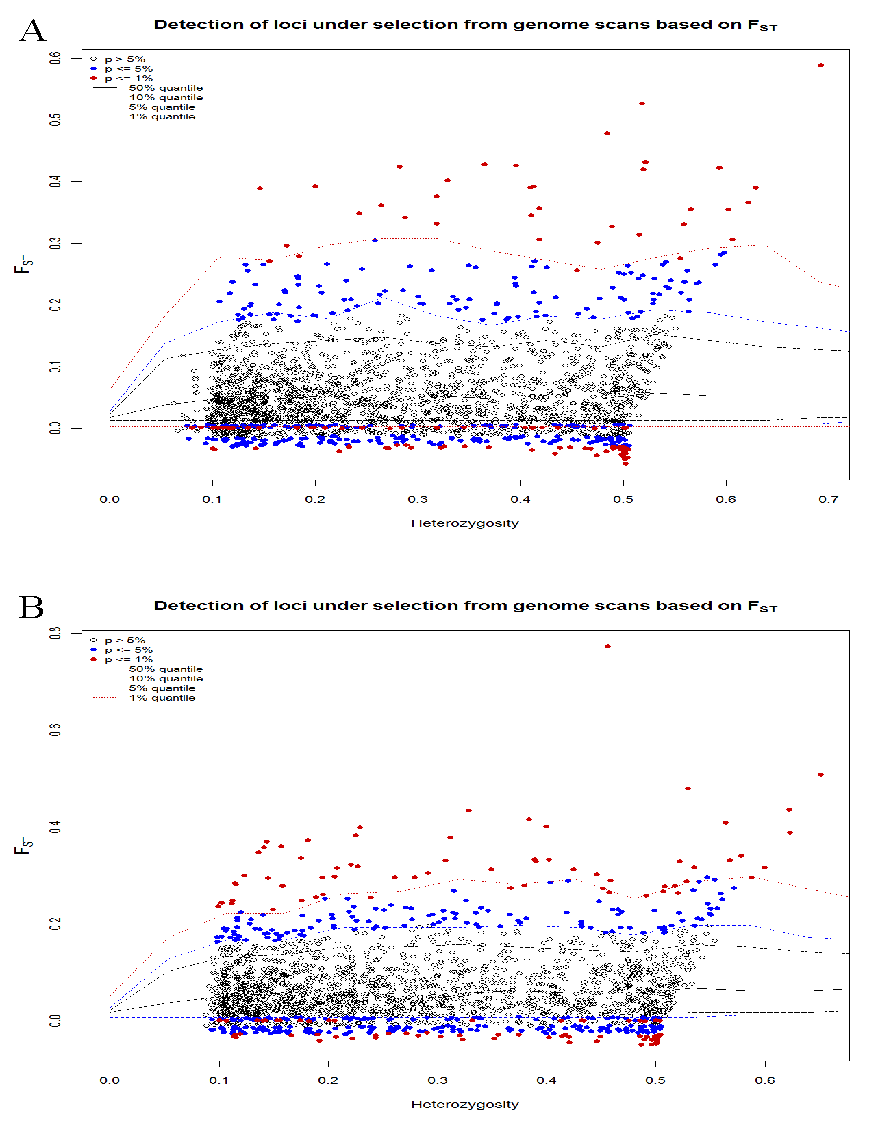


**Figure S7.** Graph of *Fst* outliers (1% outliers highlighted red and 5% outliers highlighted blue) in plot of joint heterozygosity and *Fst* values. Arlequin analyses of PE1 data from (A) library one and (B) library two using loci present in at least 75% of samples from each library and keeping the highest *Fst* SNP per RAD-tag. Results depicted here are from hierarchical model representing subpopulations nested within the coastal group or mountain-Piedmont group.


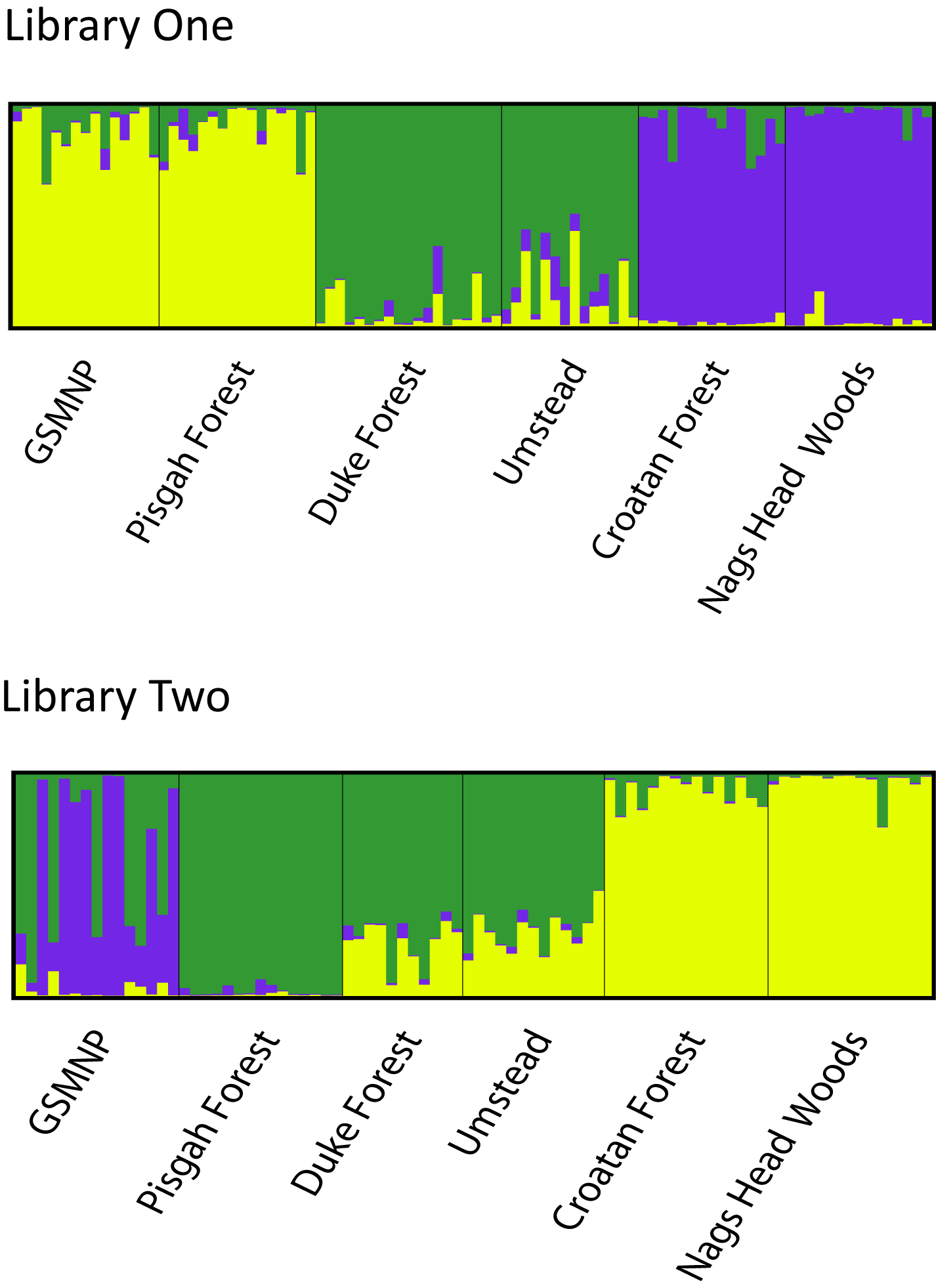


**Figure S8.** STRUCTURE results of latent factor K=3 model for library one and library two. Plots derived from ten replicate runs of STRUCTURE, a burn-in of 100,000, and 100,000 subsequent iterations. Sequence datasets parsed of correlated loci pairs via removal of multiple SNPs per RAD-tag.


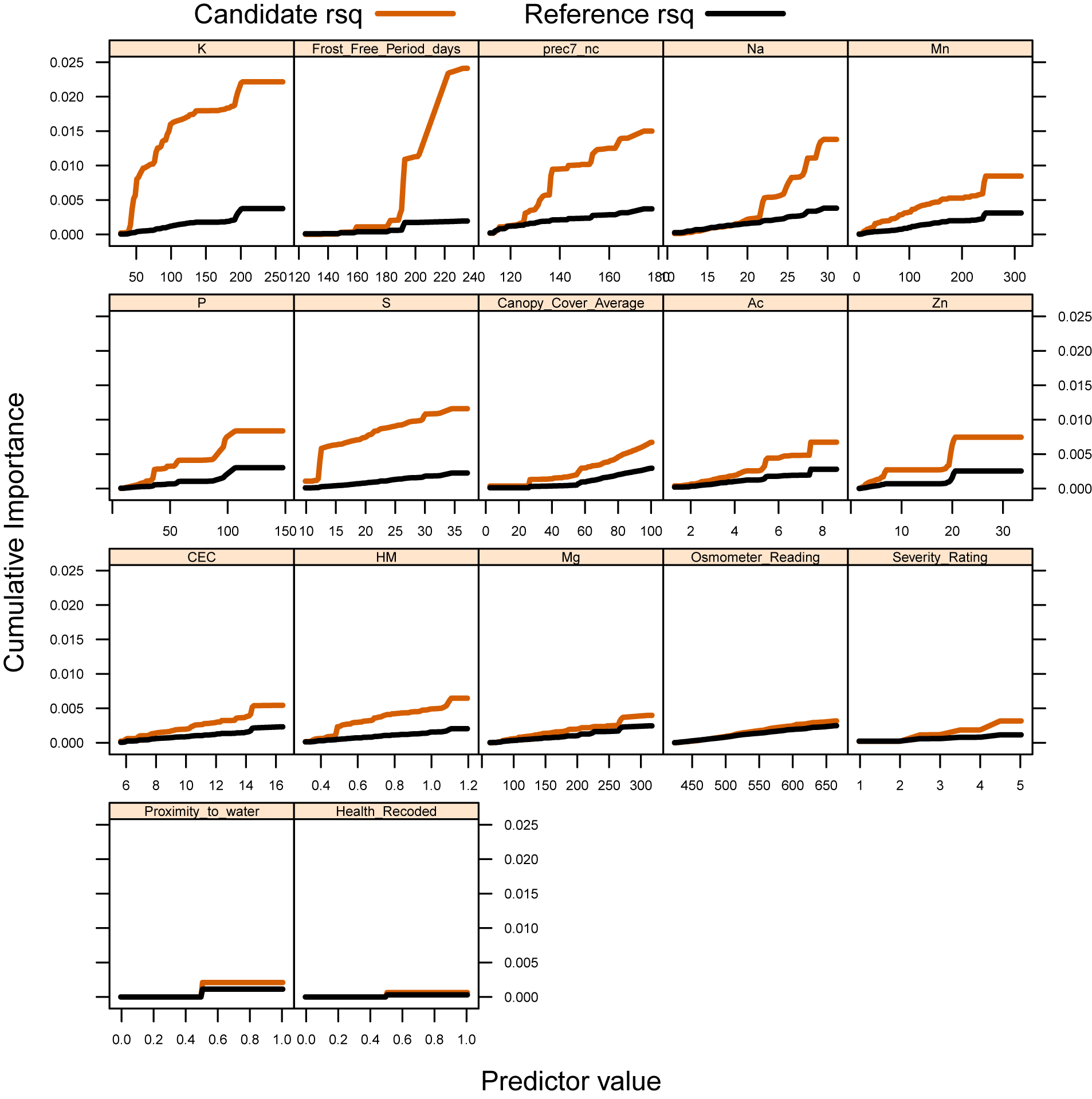


**Figure S9.** Cumulative r-squared importance along ecological gradients for gradient forest analyses. Plots ordered from top left to bottom right corresponding to the ranked combined importance in respect to gradient’s overall importance of explaining allele turnovers for the candidate dataset (vermillion) vs. the reference dataset (black).


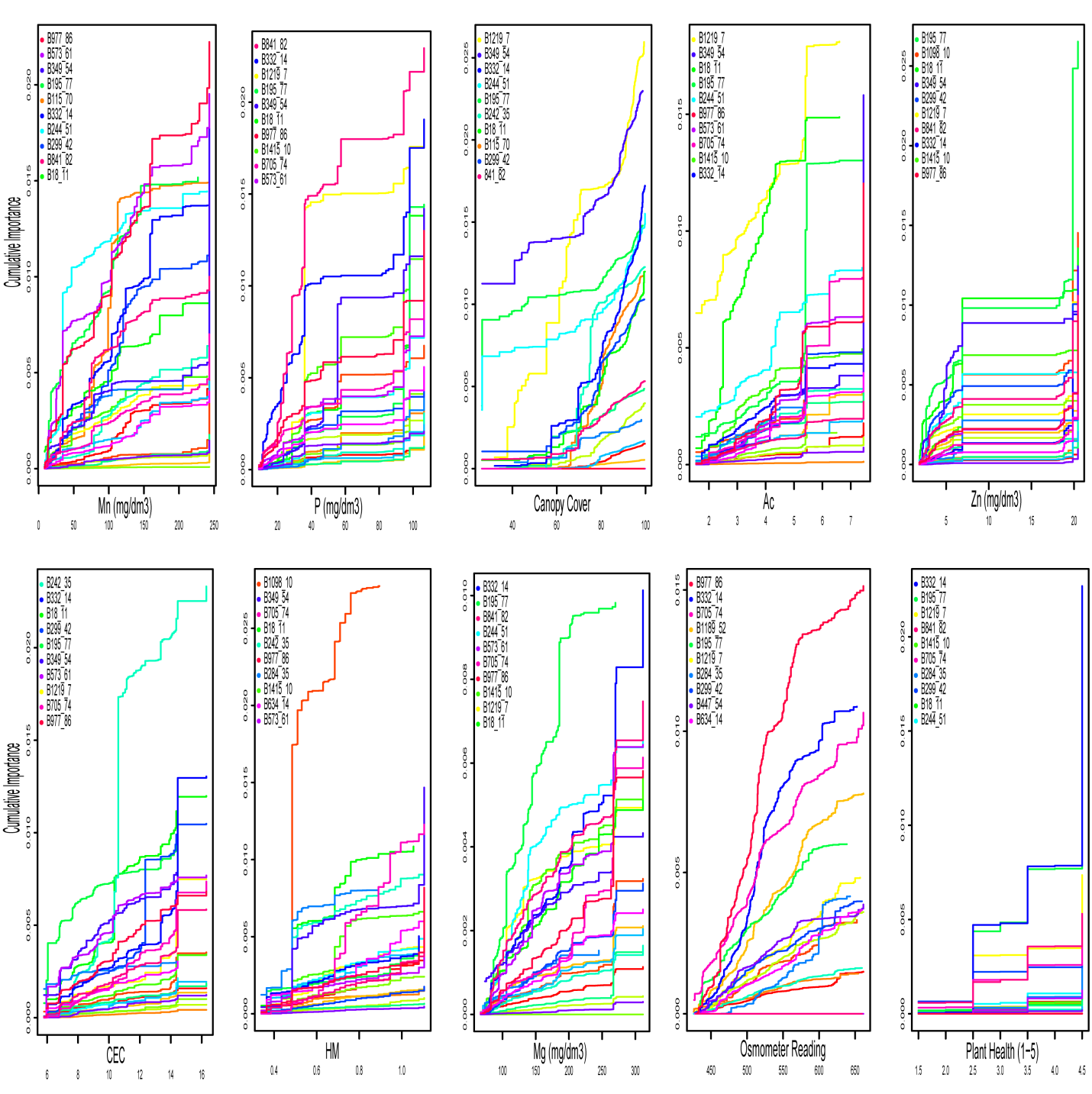


**Figure S10.** Gradient Forest plots of SNP-level compositional turnover for predictor variables other than those featured in Figure 8. Allele functions with a correlation threshold of 0.5 visualized on GF plots. Proximity to water and binary coding of plant health not listed due to zero functions passing a GF correlation threshold of 0.5. All allele functions plotted here retained signatures of being under selection when combined library of candidate and reference SNPs was reanalyzed with Arlequin, Bayescan, or LFMM.

|  | **Mountains** | | **Piedmont** | | **Coast** | |  |  |  |
| --- | --- | --- | --- | --- | --- | --- | --- | --- | --- |
| **Variable** | **Great Smokey Mountains Mean** | **Pisgah Forest Mean** | **Duke Forest Mean** | **Umstead State Park Mean** | **Croatan Forest Mean** | **Nags Head Woods Mean** | **Residuals df** | **F value*** | **One-Way ANOVA P value*** |
| **Elevation (ft)** | **3060a** | **2441b** | **444c** | **432c** | **75.4d** | **42.8d** | **173** | **596.4** | **<2.00E-16** |
| **Proximity to water (0/1)** | **0.367a** | **0.467a** | **0.2ab** | **0.414a** | **0.4a** | **1.5E-15b** | **173** | **4.789** | **3.99E-04** |
| **Canopy cover percentage** | **92.1a** | **91.8a** | **91.9a** | **96.9b** | **87.5a** | **89.3ab** | **172** | **2.899** | **0.0154** |
| **Frost free period (days)** | **159a** | **148b** | **215c** | **215c** | **235d** | **230e** | **173** | **2164** | **<2.00E-16** |
| **Mean montly rainfall (mm)** | **62.1a** | **59.2ab** | **47.9d** | **45.6e** | **55.6b** | **53.2c** | **173** | **75.19** | **<2.00E-16** |
| **Mean temp (C)** | **11.4a** | **12.4b** | **15.2c** | **15.4d** | **17.0e** | **16.7f** | **173** | **3696** | **<2.00E-16** |
| **Min Jan temp (worldclim)** | **-56.1a** | **-45.3b** | **-26.3c** | **-17.7d** | **7.55e** | **12.5f** | **170** | **9914** | **<2.00E-16** |
| **Max Jul temp (worldclim)** | **267a** | **280b** | **315c** | **316c** | **316c** | **304d** | **168** | **440.7** | **<2.00E-16** |
| **Driest month prec (worldclim)** | **111a** | **110a** | **81.7b** | **75.8c** | **79.6d** | **82e** | **170** | **55.06** | **<2.00E-16** |
| **June prec (worldclim)** | **137a** | **125a** | **99.7b** | **97c** | **128a** | **110d** | **168** | **70.16** | **<2.00E-16** |
| **July prec (worldclim)** | **144a** | **133a** | **113b** | **116c** | **174d** | **139a** | **168** | **173.9** | **<2.00E-16** |
| **Length of growing period (days)** | **211a** | **211a** | **252b** | **252b** | **293c** | **293c** | **173** | **3.71E+29** | **<2.00E-16** |
| **Phosphorous (mg/dm3)** | **22a** | **25ab** | **15.2a** | **19.5ab** | **73b** | **23.6ab** | **23** | **2.803** | **0.0405** |
| **Potassium (mg/dm3)** | **137a** | **105ab** | **76ab** | **94.2ab** | **51b** | **45.6b** | **23** | **5.082** | **0.00278** |
| **Calcium (mg/dm3)** | **980** | **499** | **1013** | **1068** | **1541** | **595** | **23** | **1.852** | **0.142** |
| **Magnesium (mg/dm3)** | **147** | **123** | **197** | **163** | **98.3** | **123** | **23** | **1.234** | **0.326** |
| **Sulfur (mg/dm3)** | **20.8ab** | **25.3a** | **20.6ab** | **18.5ab** | **19ab** | **12.2b** | **23** | **3.087** | **0.0282** |
| **Sodium (mg/dm3)** | **18.2a** | **16.3a** | **20ab** | **13.8a** | **15.3a** | **26.2b** | **23** | **6.476** | **6.80E-04** |
| **Manganese (mg/dm3)** | **86.9bc** | **40.3bc** | **175a** | **124ab** | **13.9c** | **46.8bc** | **23** | **7.854** | **1.95E-04** |
| **Copper (mg/dm3)** | **6.62** | **1.68** | **1.7** | **1.45** | **1.53** | **0.84** | **23** | **1.392** | **0.264** |
| **Zinc (mg/dm3)** | **8.98** | **3.77** | **4.1** | **3.65** | **2.2** | **3.66** | **23** | **0.878** | **0.511** |
| **Percent humic matter** | **0.753ab** | **0.88a** | **0.688ab** | **0.72ab** | **0.88a** | **0.452b** | **23** | **3.485** | **0.0172** |
| **Weight to volume soil ratio** | **0.703ab** | **0.608a** | **0.758ab** | **0.775ab** | **1bc** | **1.14c** | **23** | **6.933** | **4.43E-04** |
| **Cation exchange capacity** | **9.47** | **8.65** | **9.98** | **10** | **11.4** | **7.46** | **23** | **0.981** | **0.451** |
| **Exchangeable acidity** | **3** | **4.88** | **3.12** | **3.1** | **2.77** | **3.34** | **23** | **1.456** | **0.243** |
| **soil pH** | **5.17** | **4.43** | **4.86** | **4.9** | **5.23** | **4.52** | **23** | **1.429** | **0.251** |
| **Percent base saturation** | **64.3** | **45.3** | **67.8** | **64.8** | **69.3** | **54** | **23** | **1.297** | **0.299** |
| **Plant Health (1-5)** | **3.33a** | **3.03a** | **4.67b** | **4.48b** | **4.77b** | **4.77b** | **173** | **29.74** | **<2.00E-16** |
| **Osmolality (mmol/kg)** | **485a** | **504ab** | **518ab** | **532b** | **525b** | **532b** | **158** | **4.195** | **0.00132** |
|  |  |  |  |  |  |  |  |  |  |
| ***ANOVA values reported here. Kruskal-Wallis rank sum test interpreted if Shapiro-Wilk or Bartlett tests reported ANOVA violations (normality and homoscedasticity)** | | | | | | | | | |
| **a, b, c, d, e, and f: Different letters indicate significant differences (P<0.05) between Tukey-Kramer or Dunnet modified Tukey-Kramer (for violations of homoscedasticity) comparisons.** | | | | | | | | | |

**Table S1 Appendix.** Matrix of population-level statistics of environmental-functional variables.

|  |  |  | *Percentage* |  |  |  |
| --- | --- | --- | --- | --- | --- | --- |
|  | *Variation source* | *d.f.* | *of variation* | *P-value* |  |  |
|  |  |  |  |  |  |  |
| *Library One* |  |  |  |  |  |  |
| *Dataset* |  |  |  |  |  |  |
|  | *Among groups* | 2 | 2.33 | 0.06716 |  |  |
|  |  |  |  |  |  |  |
|  | *Among populations* | 3 | 1.69 | <0.0001 |  |  |
|  | *within groups* |  |  |  |  |  |
|  |  |  |  |  |  |  |
|  | *Among individuals* |  |  |  |  |  |
|  | *within populations* | 88 | 2.33 | 0.08580 |  |  |
|  |  |  |  |  |  |  |
|  | *Within* |  |  |  |  |  |
|  | *Individuals* | 94 | 93.65 | 0.00042 |  |  |
|  |  |  |  |  |  |  |
| *Library Two* |  |  |  |  |  |  |
| *Dataset* |  |  |  |  |  |  |
|  | *Among groups* | 2 | 2.9 | 0.06571 |  |  |
|  |  |  |  |  |  |  |
|  | *Among populations* | 3 | 2.73 | <0.0001 |  |  |
|  | *within groups* |  |  |  |  |  |
|  |  |  |  |  |  |  |
|  | *Among individuals* | 78 | 5.42 | 0.00120 |  |  |
|  | *within populations* |  |  |  |  |  |
|  |  |  |  |  |  |  |
|  | *Within* | 84 | 88.95 | <0.0001 |  |  |
|  | *Individuals* |  |  |  |  |  |

**Table S2.** AMOVA results from independent analyses of library one and two datasets. Three groups represented are the coastal group, Piedmont group, and Mountain group. 50,000 permutations ran in Arlequin for test of significance.

**Table S3.** Mantel and partial mantel results from analysis of loci genotyped consistently across library one and two. Geographic distance was used in partial mantel tests to partition and control for possible covariation with genetic distance (linearized *Fst* of sites). Reference (1171 SNPs) and candidate (43 SNPs) datasets reported. Ac, CEC, and HM abbreviations represent exchangeable acidity, cation exchange capacity and humic matter, respectively. Prec7 represents the monthly average of precipitation in July. All other abbreviations stand for periodic elements. Using 10,000 permutations specified by R functions (ecodist package), each p-value is based on the null hypothesis that the Mantel R is less than zero.

**Table S4.** The proportion of total adaptive variation captured by current pilot study compared to samples being prepared for study of the entire *C. florida* range. 19 bioclim variables at 30 arc-sec (~1km) resolution were downloaded (Hijmans et al 2005) to compare samples in this pilot study with samples from broader study of the species range. Using only variables with a VIF score below 10 after step-wise removal of variables, principal component analysis was done to determine sample scores corresponding to the first eight principal components (PC). We determined the range of PC scores for the pilot study’s samples and all total samples. In addition, we partitioned total variance to determine the proportion of variance (environment derived PC scores) captured by samples in the pilot study.
